# Water-stress induced downsizing of light-harvesting antenna complex protects developing rice seedlings from photo-oxidative damage

**DOI:** 10.1038/s41598-017-14419-4

**Published:** 2018-04-13

**Authors:** Vijay K. Dalal, Baishnab C. Tripathy

**Affiliations:** School of Life Sciences, Jawaharlal Nehru University, New Delhi, 110067 India

## Abstract

The impact of water-stress on chloroplast development was studied by applying polyethylene glycol 6000 to the roots of 5-day-old etiolated rice (*Oryza sativa*) seedlings that were subsequently illuminated up to 72 h. Chloroplast development in drought environment led to down-regulation of light-harvesting Chl-proteins. Photosynthetic proteins of Photosystem II (PSII) and oxygen evolving complex i.e., Cytb559, OEC16, OEC23 and OEC33 as well as those of PSI such as PSI-III, PSI-V, and PSI-VI, decreased in abundance. Consequently, due to reduced light absorption by antennae, the electron transport rates of PSII and PSI decreased by 55% and 25% respectively. Further, seedling development in stress condition led to a decline in the ratio of variable (Fv) to maximum (Fm) Chl a fluorescence, as well in the quantum yield of PSII photochemistry. Addition of Mg^2+^ to the thylakoid membranes suggested that Mg^2+^-induced grana stacking was not affected by water deficit. Proteomic analysis revealed the down-regulation of proteins involved in electron transport and in carbon reduction reactions, and up-regulation of antioxidative enzymes. Our results demonstrate that developing seedlings under water deficit could downsize their light-harvesting capacity and components of photosynthetic apparatus to prevent photo-oxidative stress, excess ROS generation and membrane lipid peroxidation.

## Introduction

Abiotic challenges faced by plants are water-stress, salt-stress, high or low temperature-stress, metal-toxicity, nutrient-deprivation and light-stress (for a background on abiotic stress and plants, see Pareek *et al*.^1^). Among these stresses, water-stress (drought) is the most widespread on our Earth. Seedling development, flowering and grain filling are important stages in the life cycle of crop plants, leading to yield losses under stress conditions^2^.

Acclimation responses of mature plants to drought include closure of stomata, hardening of cuticle and cell wall, increased synthesis of osmolytes and antioxidants, altered cell homeostasis and metabolomes, and decreased photosynthesis^3,4^. Photosynthesis is a vital process that is affected by drought, which induces a decrease in the rate of carbon fixation due to reduced photosynthetic electron transport as well as carbon assimilation; this, in turn, results in decreased yield^3,5^. Photosynthetic responses to drought are highly complex; they depend on the intensity and the duration of stress, as well as on the developmental stage of plants.

Carbon assimilation in plants, under water-stress, is mostly affected by lowered CO_2_ availability, caused by stomatal closure and lower mesophyll conductance^6^, as well as other non-stomatal limitations^3,7^. Miyazawa *et al*. found that mesophyll conductance during drought is reduced by a disturbance in the functioning of aquaorins, which were involved in CO_2_ diffusion to the site of carboxylation by Rubisco^8^.

Prolonged exposure of mature well developed plants to water-stress results in decreased carboxylation efficiency of Rubisco and reduction in the activity of various other enzymes of the carbon fixation pathway^3,9^. Physiological, biochemical and proteomic studies have shown that water-stress reduces protein abundance of carbon reduction cycle enzymes, such as Rubisco, Fructose 1,6-bisphosphate aldolase (FBA; EC 4.1.2.13), Sedoheptulose-1, 7-bisphophatase (EC 3.1.3.37), Triose Phosphate Isomerase (TPI; EC 5.3.1.1), and Phosphoglycerate kinase (PGK; EC 2.7.2.3)^10–16^. However, it is interesting to note that in some studies Rubisco content was not severely affected, and photosynthetic CO_2_ fixation was mostly restored at Ci equivalent to that under control conditions—as was shown by applying high external CO_2_ concentrations^17–20^.

Water-stress is known to affect electron transport from water to NADP. Water-stress has been shown to damage the oxygen-evolving complex of PSII^21^, as well as both the PSII and PSI reaction centers^14,22–24^. Further, 30% PEG 6000 treatment of mature rice plants results in a reduction of photosynthetic electron transport^25^. Also, water-stress-induced stomatal closure and decreased stomatal conductance limit CO_2_ availability that results in diverting electrons from the photosynthetic electron transport chain to molecular oxygen generating ROS (O_2_^−^) at the end of PS I^26,27^. At the same time, energy transfer from  ^3^P680* (triplet state of PSII reaction center chlorophyll *a*, in its excited state) and even from antenna chlorophylls (Chl), in their triplet state, to oxygen generates singlet oxygen (^1^O_2_) that often damages thylakoid membranes^27–31^.

Rice seeds germinate beneath the soil and the seedlings grow in near-darkness till they emerge to the surface (skotomorphogenesis). During skotomorphogenesis, seedlings do not synthesize Chl, since the Chl biosynthesis pathway enzyme protochlorophyllide oxidoreductase (POR) (EC 1.3.33.1) requires light to photo-transform Pchlide to Chlide^32^. Therefore, in rice, the differentiation of etioplast to chloroplast does not take place in darkness. Upon exposure to light, as the seedlings grow, Chl biosynthesis and the associated greening process transform the etioplasts into chloroplasts^33^. These developing seedlings are very often exposed to drought, due to scarcity of rainfall. These developing seedlings are able to withstand water-stress for a long time^2^ and rebound upon rain fall. Therefore, it is important to understand its underlying mechanism of tolerance to water-stress.

Photosynthetic responses of mature plants and developing greening seedlings to water-stress are fundamentally different. In mature leaves, functional photosynthetic complexes are already formed and water-stress induces generation of ROS due to excess light absorption, which affect the photosynthetic apparatus. However, in water-stressed developing seedlings, there is the possibility to down-regulate Chl biosynthesis and downsize the synthesis and assembly of light-harvesting complexes of PSI and PSII, and to adapt plants not  to absorb excess light, which is harmful. In our previous study^33^, we have shown that PEG-induced water-stress during early rice seedling development minimizes Chl biosynthesis. Active photosynthesis complexes are formed by the assembly of apo-proteins and incoporation of chlorophyll in them^34^. Therefore, reduced Chl synthesis in water-stressed developing seedlings is likely to affect chloroplast biogenesis. PEG-6000 has long been utilized as a reliable osmoticum for the simulation and constitutive maintenance of water-stress in plants under laboratory conditions, due to its property to act as a non-penetrating osmotic agent that decreases water potential of the cells^35,36^. To understand the mechanism of relative tolerance of developing seedling to drought, we have studied the impact of water-stress on the photosynthetic apparatus during early seedling growth, as they emerge from skotomorphogenesis beneath the soil to photomorphogenesis stage above the soil.

## Results

To understand the impact of water-stress on chloroplast biogenesis during the early photomorphogenesis, 5-d-old etiolated rice seedlings were treated with polyethylene glycol (PEG 6000) for 16 hours prior to their transfer to continuous cool-white fluorescent (plus incandescent) light (100 µmol photons m^−2^ s^−1^). In this paper, we have analyzed the structure and function of chloroplasts, ROS production, and associated processes to assess the role of water-stress on early seedling development.

### Impact of Water-Stress on Chloroplast Development

#### Chl *a* fluorescence measurements

**Figure 1:**
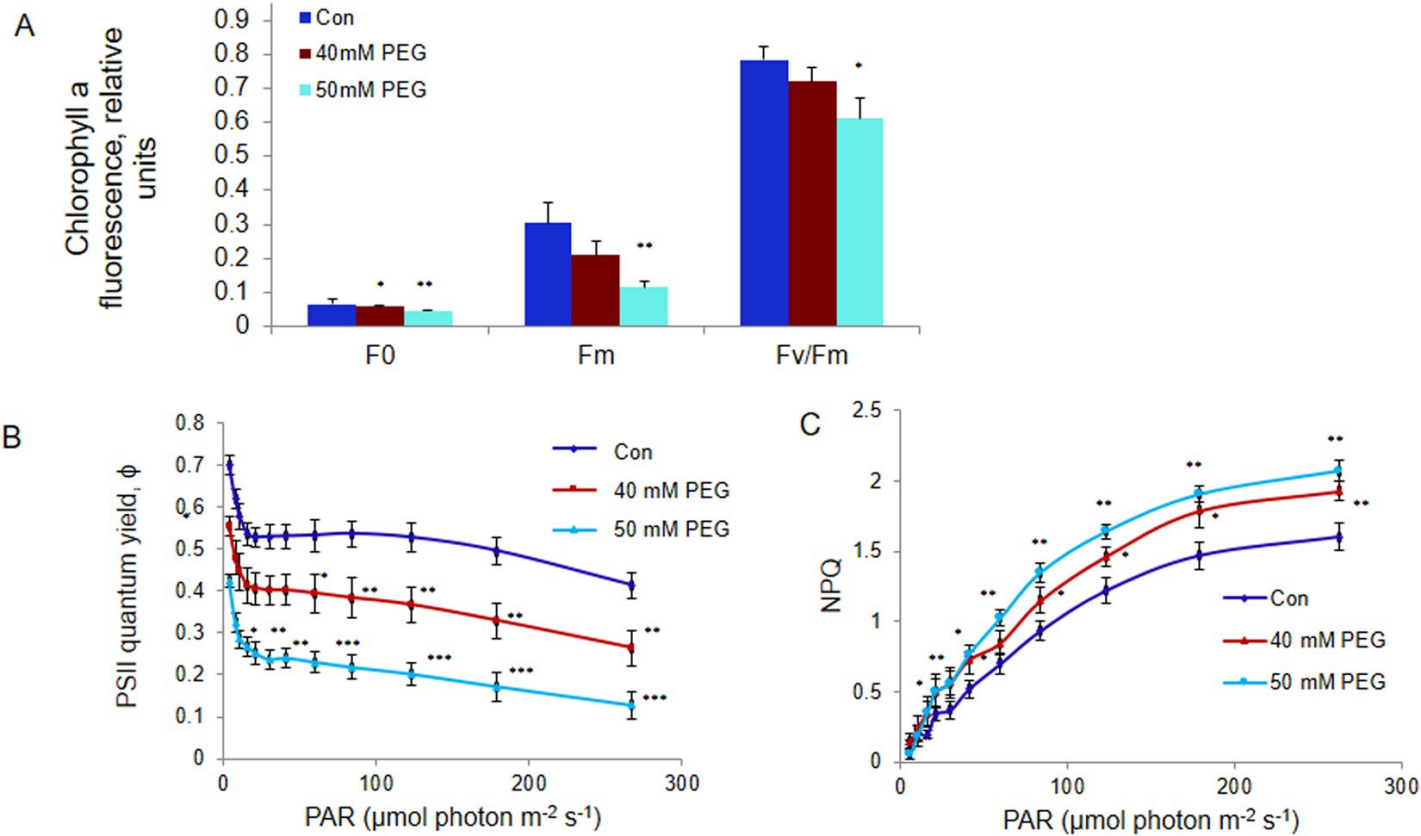
Chlorophyll *a* fluorescence measurements of control and water-stressed rice seedlings after 72 h of greening. (**A**) Fo, Fm, Fv/Fm = (Fm − Fo)/Fm, (**B**) “Operating” quantum yield (Φ) of PSII and (**C**) Non-photochemical quenching (NPQ). Five-day old etiolated seedlings were treated with 40 mM (or 50 mM) PEG 6000 dissolved in half strength MS nutrient soln., 16 h prior to their illumination with cool white fluorescent + incandescent light (100 μmol photons m^−2^ s^−1^), at 28 °C. Chl *a* fluorescence based parameters were measured with a PAM 2100 fluorometer. “Operating” PSII quantum yield (Φ) and NPQ were calculated as (Fm′ − Ft)/Fm′ and (Fm-Fm′)/Fm′ respectively. Each data point is the average of three replicates. The error bars represent standard deviations (SD). ANOVA: *p < 0.05; **p < 0.01; and *** p < *0.001*.

Fo, Fm and Fv/Fm ratio were monitored after water-stress treatment of etiolated rice seedlings for 72 h with 40 mM and 50 mM PEG, in the presence of light (100 μmol photons m^−2^ s^−1^). Seedlings were kept for 20 min in darkness (see Materials and Methods) before the initial (Fo), and the maximum (Fm), fluorescence was measured. The minimum fluorescence Fo decreased by 11% and 21% in the seedlings treated with 40 and 50 mM PEG compared to the controls, while Fm decreased by 32% and 63% respectively (see Fig. 1A).

The ratio Fv/Fm = (Fm − Fo)/Fm, a “proxy” of the maximum quantum yield of PSII photochemistry^37^, declined in water-stressed samples; Fv/Fm calculated after 72 h of greening decreased by 10% and 22% in 40 and 50 mM PEG-treated seedlings (Fig. 1A).

### Quantum yield of PSII (ϕPSII) and non-photochemical quenching (NPQ) during 300 s illumination with actinic light

As expected, ϕPSII declined at higher light intensity. Compared to the controls, ϕPSII, at the highest light intensity (~275 µmol photons m^−2^ s^−1^), was reduced by 33% and 58% in samples treated with 40 mM and 50 mM PEG (Fig. 1B). On the other hand, the NPQ ((Fm − Fm′)/Fm′) increased in response to increase in light intensity. It was higher in 40 mM and 50 mM PEG-treated seedlings than in controls by 21% and 29% under the highest (~275 µmol photons m^−2^ s^−1^) light intensity used (Fig. 1C).

#### Room temperature (298K) Chl a fluorescence spectra

**Figure 2:**
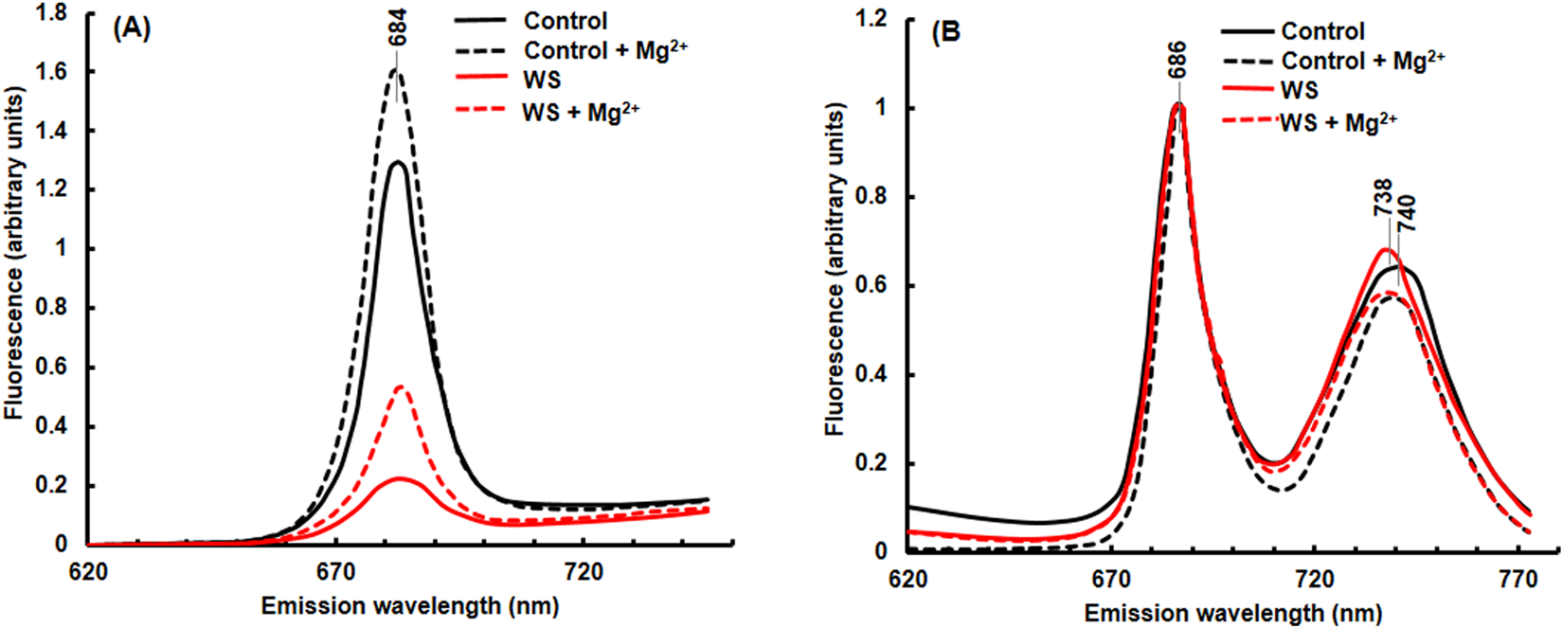
Fluorescence emission spectra of thylakoid membranes isolated from leaves of control and water-stressed rice seedlings. (**A**) Room temperature and (**B**) Low temperature (77K) fluorescence spectra (E440) of thylakoid membranes (3 μg Chl) suspended in 5 mM Hepes-NaOH buffer containing 0 or 4 mM MgCl_2_ (pH 7.5), after 72 h of stress treatment. Seedlings were treated as in Fig. 1. Fluorescence emission spectra were recorded in ratio mode in a photon counting SLM-AMINCO 8000 spectrofluorometer. For 77 K spectral measurements, excitation and emission slit widths were set at 4 nm. For room temperature spectra, the excitation and emission slit widths were set at 8 nm and 4 nm, respectively. The room temperature spectra were corrected for photomultiplier tube response. Rhodamine B was used in the reference channel as a quantum counter. A tetraphenylbutadiene (TPD) block was used to adjust the voltage to 20000 counts per second in the sample as well as in the reference channels at excitation and emission wavelengths of 348 nm and 422 nm, respectively. Fluorescence spectra were measured three times and identical results were obtained.

Chl *a* fluorescence emission spectrum of thylakoid membranes isolated (after 72 h greening) had a peak at 684 nm; this is mostly, from PSII antenna^38^. Upon the addition of Mg^2+^ in the suspension medium, this peak increased in the control as well as in the water-stressed samples (Fig. 2A). In water-stressed samples, the fluorescence intensity at 684 nm was substantially lower both in the presence and the absence of Mg^2+^, compared to the controls.

#### Low temperature Chl a fluorescence spectra

Low temperature (77 K) fluorescence emission spectra monitored after 72 h of light exposure are shown in Fig. 2B. Thylakoid membranes of control samples, suspended in a low salt medium (0 mM Mg^2+^), had a peak at 686 nm due to PSII and at 740 nm due to PSI^38^; usually, PSII shows two emission bands at ~684 nm (from light- harvesting complex) and at 695 nm (from CP47), although a single peak has been observed in some cases (e.g. in *Gonyaulax polyedra*)^39^. Fluorescence emission spectra, shown here, were normalized at 686 nm. In water-stressed samples, the emission peak of PSI shifted from 740 nm to 738 nm. Upon addition of Mg^2+^ (4mM), the PSI fluorescence emission at 740 nm (normalized at 686 nm; PSII) decreased in both the control and the water-stressed samples.

### PSII, PSI and the whole chain electron transport in isolated thylakoid membranes

To probe further results obtained from Chl *a* fluorescence measurements, we monitored partial PSII and PSI electron transport reactions, as well as the whole photosynthetic electron transport chain in thylakoid membranes isolated from control and water-stressed seedlings.

#### PSII activity

**Figure 3:**
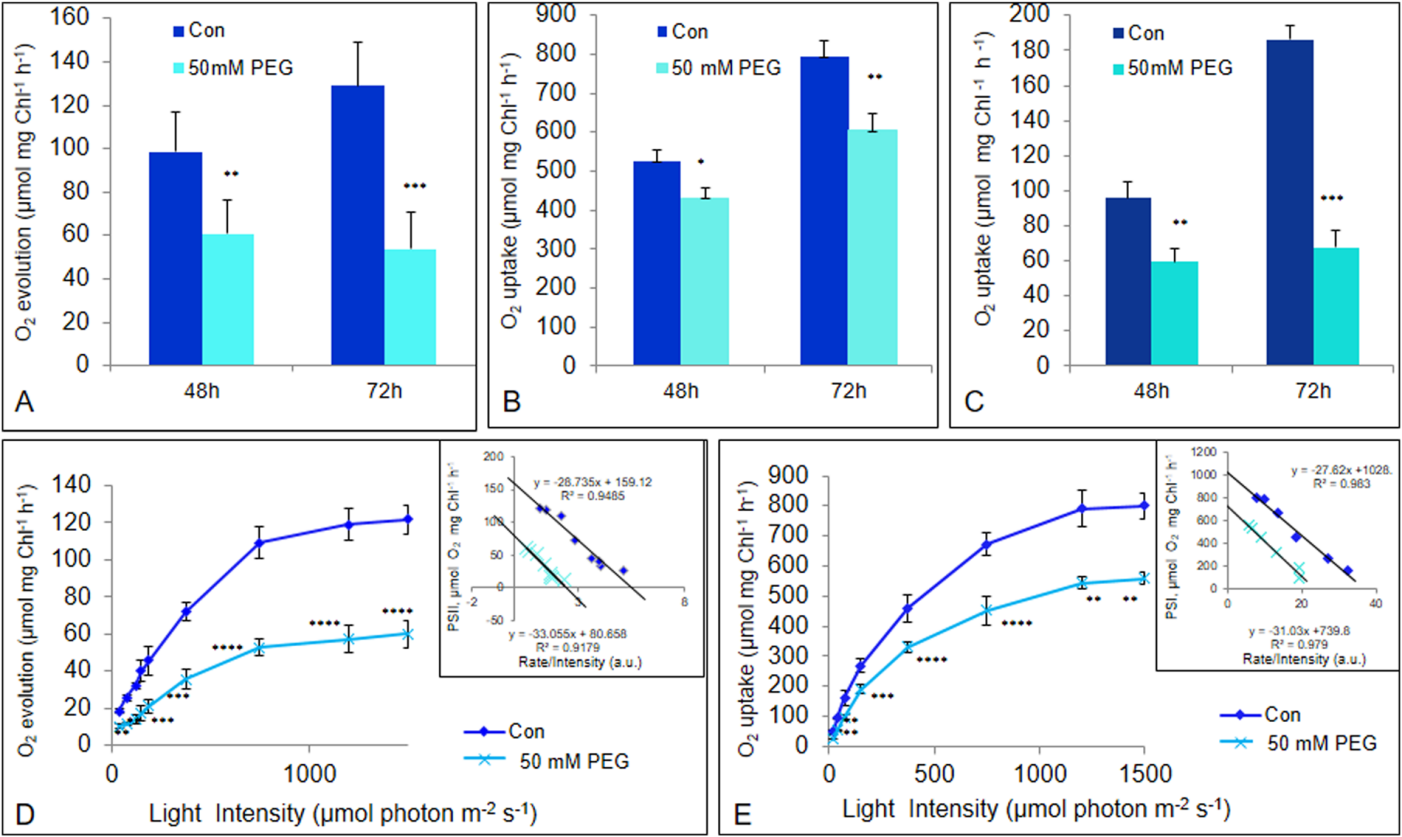
Photosynthetic polarographic measurements of thylakoid membranes isolated from control and water-stressed rice seedlings. (**A**) PSII, (**B**) PSI and (**C**) whole chain activities after 48 h and 72 h of greening, and light saturation curve of (**D**) PSII and (**E**) PSI reactions after 72 h of greening in thylakoid membranes isolated from control and water-stressed seedlings. Seedlings were treated essentially as in Fig. 1. Oxygen evolution/uptake by thylakoid membranes was measured by Oxy Lab, Hansatech. Thylakoids, equivalent to 20 µg of Chl, were used for each reaction. PSII, PSI and whole chain activities were measured as electron transport from H_2_O to p-phenylenediamine (PD), Ascorbate/DCIP couple to methylviologen (MV) and H_2_O to MV, respectively. For measurement of light saturation curve, different light intensities were obtained from a tungsten lamp using neutral density filters. Each data point is the average of three replicates. The error bars represent SD. ANOVA: *p < 0.05; **p < 0.01; and *** p < *0.001*.

The partial PSII electron transport, which was measured polarographically as light-driven electron transport from H_2_O to phenylenediamine, increased as the greening process of etiolated control seedlings progressed (Fig. 3A). Further, in 50 mM PEG-treated seedlings, PSII activity was reduced by 35% and 55% after 48 h and 72 h of greening compared to that in the controls.

#### PSI activity

The partial PSI electron transport, which was measured polarographically as light-driven electron transport from ascorbate/DCIP to methylviologen, increased in response to chloroplast development during light exposure. Similar to PSII, PSI electron transport decreased in water-stressed seedlings, compared to controls, but to a lower extent than that of PSII: i.e., by 20% and 25% after 48 h and 72 h of chloroplast biogenesis (Fig. 3B).

#### The whole (photosynthetic) electron transport chain

The whole chain electron transport through PSII and PSI, which was measured polarographically as light-driven electron transport from H_2_O to methylviologen, was reduced by 35% after 48 h and 65% after 72 h of greening in stressed seedlings compared to control (Fig. 3C).

### Light saturation curves of PSII and PSI electron transport reactions

#### PSII

To further ascertain if the inhibition of PSII reaction was due to reduction in the quantum yield of PSII photochemistry measured in limiting light intensities or it was in light saturated electron transport, we measured the rate of PSII reaction as a function of different light intensities, using thylakoid membranes isolated from the control as well as from water-stressed seedlings, after 72 h of greening. The dependence of PSII activity on light intensity showed typical saturation kinetics (Fig. 3D). Both the initial slope at limiting light intensities as well as light-saturated electron transport, were affected in PEG-treated seedlings. As compared to the control thylakoids, the percent inhibition of PSII reaction in water-stressed thylakoids was almost constant (nearly 50%) at all the light intensities used (Fig. 3D). The Eadie plot (i.e., the rate of oxygen evolution *vs* rate of oxygen evolution/light intensity in terms of % saturation^40^ showed a straight line characterized by the equation y = −28.73x + 159.1 (R^2^ = 0.948) for the control seedlings and y = −33.05x + 80.65 (R^2^ = 0.917) for the water-stressed seedlings (insets in Fig. 3D). Both the intercepts on the abscissa and on the ordinate were reduced by nearly 50%.

#### PSI

In the PSI case, both the initial slope of electron transport rate at limiting light intensity as well as high light intensity i.e., for saturated electron transport rate, were reduced almost equally by ~30% (Fig. 3E). The Eadie plot showed a straight line with an equation y = −27.62x + 1028 (R^2^ = 0.983) for the control seedlings and y = −31.03x + 739.8 (R^2^ = 0.979) for the water-stressed seedlings (inset Fig. 3E). Intercepts on the abscissa and on the ordinates were reduced by nearly 30%.

### Immunoblot analysis of photosynthetic proteins

To understand the mechanism of water-stress induced down-regulation of photosynthesis during the development of the photosynthetic apparatus in the rice seedlings, we performed immunoblot analysis of proteins involved in PSII, PSI and the inter-system electron transfer.

#### PSII

**Figure 4:**
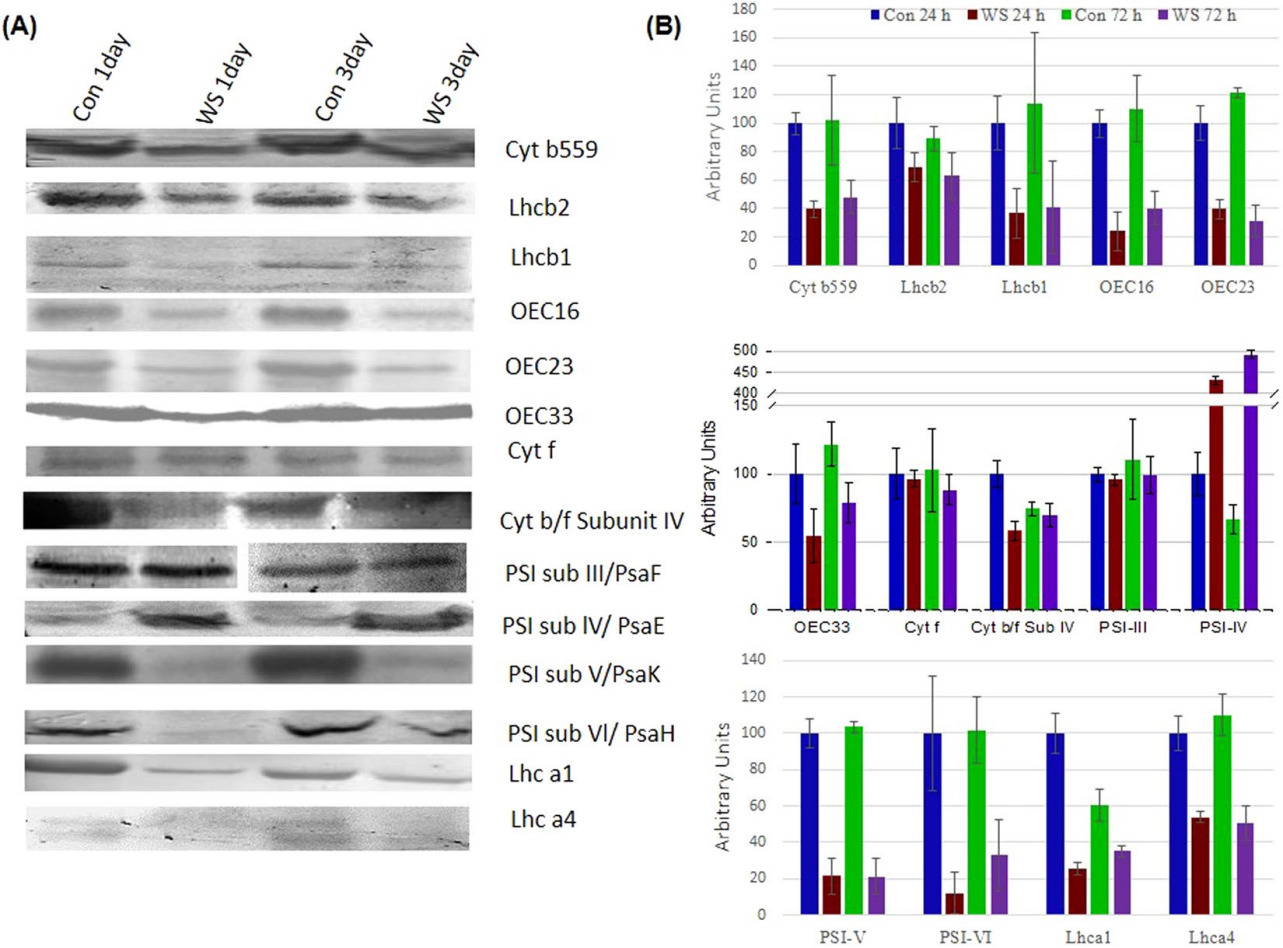
Immunoblot analysis of photosynthetic proteins. (**A**) Immunoblot and (**B**) Quantitation of immunoblots; Seedlings were treated as described in Fig. 1. Thylakoid membranes were isolated from control and water-stressed rice seedlings after 24 h and 72 h of greening. Equal amount of protein (20 µg) was separated on 12.5 % SDS-PAGE. After transfer to nitrocellulose membranes, the blotted bands were immune-detected with specific antibodies for each protein and subsequently visualized utilizing alkaline phosphatase labeled anti-IgG antibodies. Quantitation of immunoblots was performed with ImageJ and is represented as percent of control (24 h) values. In immunoblot image of PSI sub III, intervening lines have been removed (white gap). Error bars represent SD of three independent replicates.

Most of proteins/pigment-protein complexes of PSII increased upon illumination of the seedlings. The abundance of cyt b559, an intrinsic membrane protein intimately associated with PSII reaction center, declined by 58% and 52% from that in the controls, in the developing rice seedlings after 24 and 72 h of water-stress treatment (Fig. 4). Further, light-harvesting pigment-protein complexes associated with PSII i.e., Lhcb2 and Lhcb1, were reduced by 30% and 63.4% after 24 h of water-stress treatment, and by 30% and 64% after 72 h treatment (Fig. 4).

#### Oxygen Evolving Complex, OEC

Most of the oxygen evolving complex proteins were severely reduced in the rice seedlings subjected to water-stress during chloroplast development. The OEC16 and OEC23 were reduced by ~75% and 66% respectively in water-stressed seedlings (Fig. 4) after 24 h of stress. Similarly, at this time point, other major OEC protein i.e., OEC33 was reduced by ~50% (Fig. 4).

#### Cytochrome b_6_/f

The protein abundance of cyt f, as well as the subunit IV of cyt b_6_/f complex declined by 4% and 15%, and by 40% and 7% after 24 h and 72 h of water-stress respectively during chloroplast biogenesis (Fig. 4).

#### PSI

In water-stressed seedlings, the protein abundance of the PSI subunit III (PsaF, 22 kD) was reduced by ~5%, while protein expression of the subunit V (PsaK, 17 kD) was severely reduced by ~80% after 24 h/72 h treatment (Fig. 4). Further, the PSI subunit VI (PsaH, 11 kD) was reduced by 88% and 67%, due to 24 h and 72 h water-stress. In contrast, PSI subunit IV (PsaE, 11 kD) had increased by 4 and 5 fold in water-stressed seedlings (Fig. 4). The PSI antenna proteins Lhca1 and Lhca4 were reduced by 75% and 46% after 24 h of water-stress treatment, and by 42% and 55% in seedlings water-stressed for 72 h.

### Ultrastructure of chloroplast thylakoids

**Figure 5:**
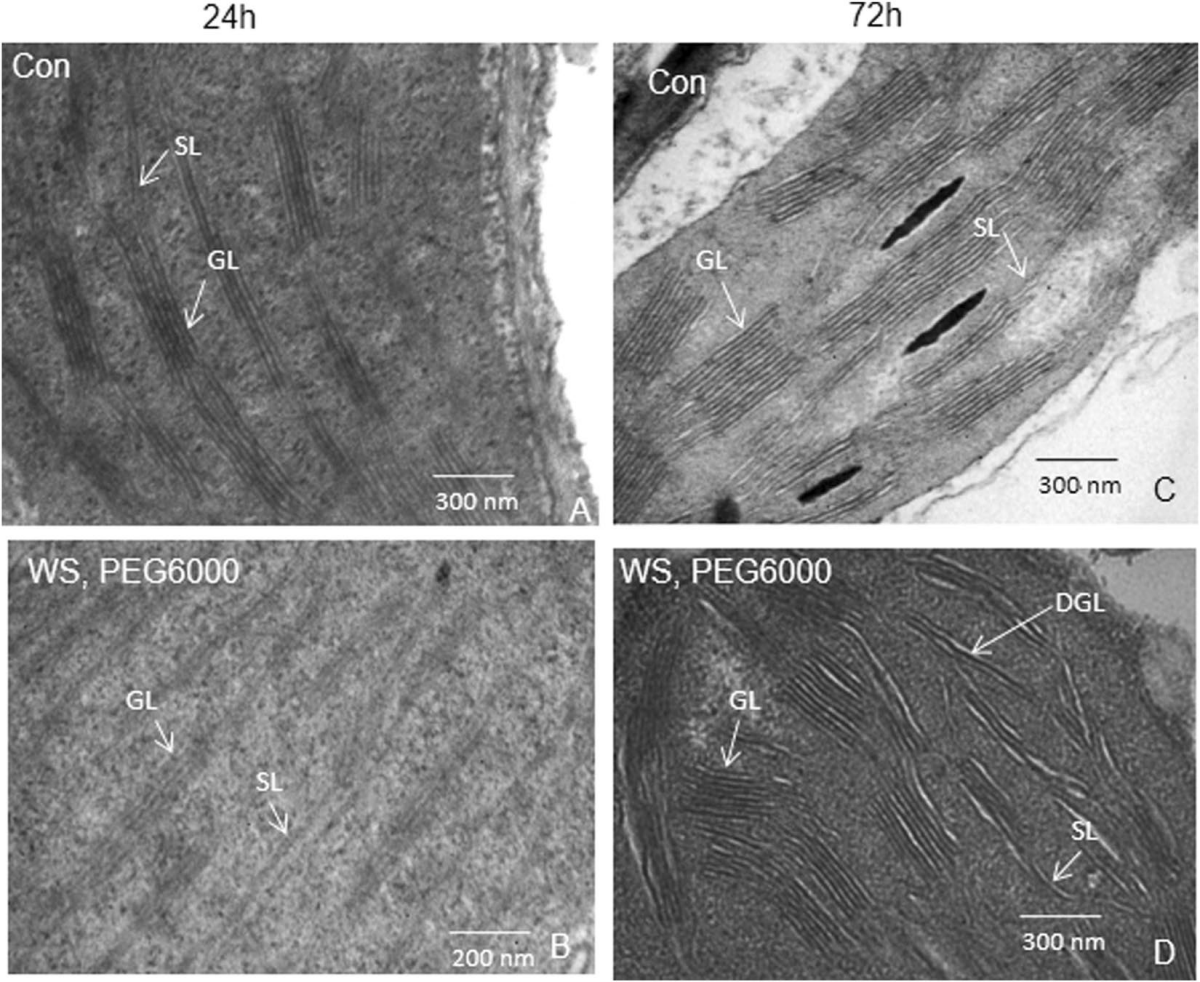
Ultrastructure of chloroplasts. Transmission electron micrograph depicting the ultrastructure of chloroplast was performed in control and water-stressed rice seedlings, after (**A** and **B**) 24 h and (**C** and **D**) 72 h of greening. Seedlings were treated as described in Fig. 1. Glutaraldehyde fixed tissues were dehydrated with acetone, cleared with epoxy propane or xylene and infiltrated with araldite. Araldite was polymerized at 50 °C for 12–24 h and then at 60 °C for 24–48 h. Ultrathin sections were cut, stained in saturated uranyl acetate in 50% ethanol for 10–15 min, washed in 50% ethanol and distilled water, and viewed in a Transmission Electron Microscope. The electron micrograph of chloroplasts were performed three times and representative figures are displayed. Scale bar is 200/300 nm. GL-grana lamellae, SL-stroma lamellae, DGL-dis-integrated grana lamellae.

After 24–72 h of greening, both thylakoids and grana were well developed and contained starch granules in control samples. However, chloroplasts in the water-stressed seedlings had swollen thylakoids, disintegrated granal organization (mostly after 72 h) and fewer starch granules (Fig. 5).

### H_2_O_2_ content

**Figure 6:**
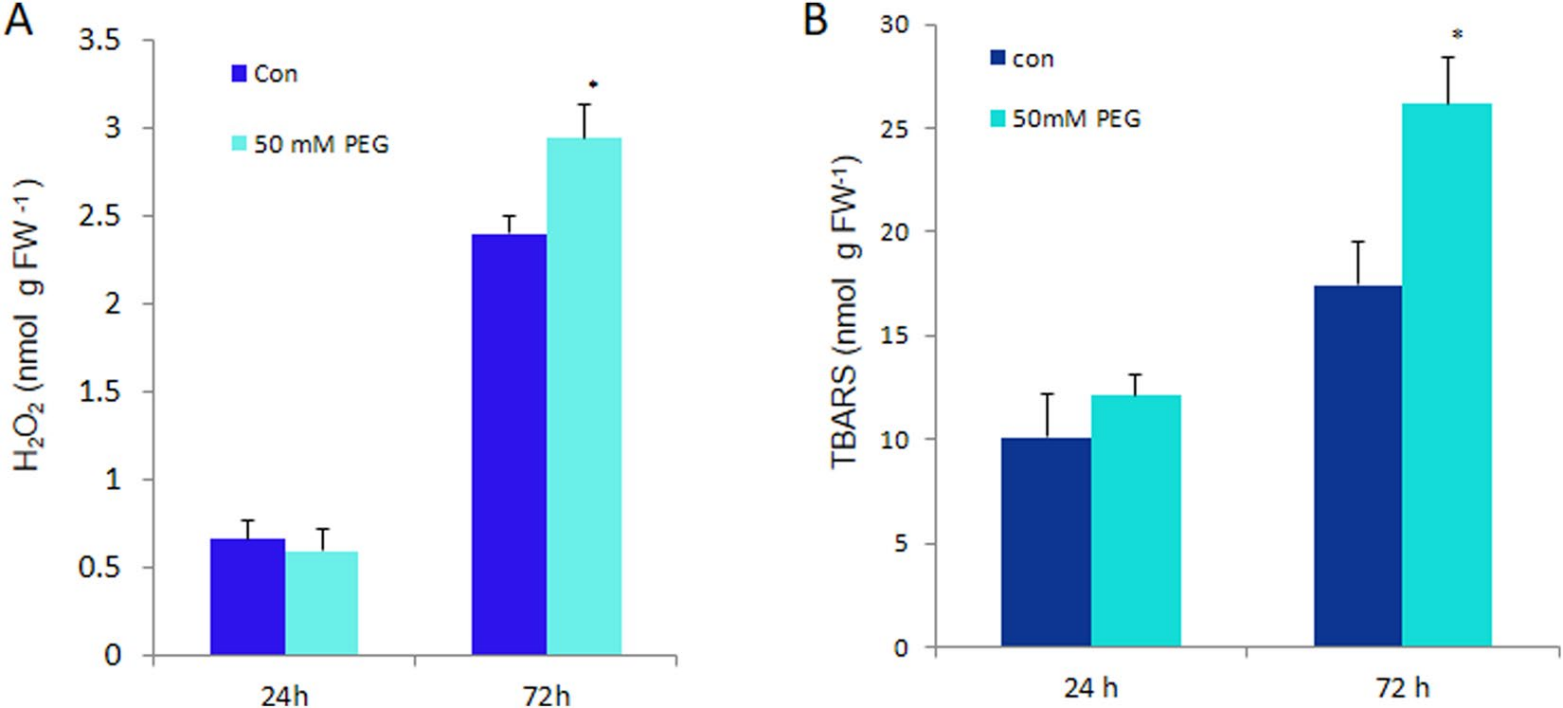
Antioxidative response of seedlings. (**A**) H_2_O_2_ content and (**B**) MDA content in control (Con) and stressed (50 mM PEG) rice seedlings after 24 h and 72 h of greening. Seedlings were treated as in Fig. 1. H_2_O_2_ was measured by absorbance at 240 nm, against a standard curve and MDA was measured as TBARS equivalents. Leaf tissue (200 mg) was homogenized in 20% TCA solution with and without 0.25% TBA, boiled and centrifuged. Absorptions at 440, 532 and 600 nm were measured from supernatant. Unspecific absorption contribution from without-TBA solution was deducted appropriately (see M&M) from sample values. The error bar represents SD of three replicates. ANOVA: *p < 0.05; **p < 0.01; and ***p < *0.001*.

H_2_O_2_ content remained approximately the same in both water-stressed and the control rice seedlings, after 24 h of greening (Fig. 6A); however, after 72 h of greening, H_2_O_2_ content increased by 23% as compared to control samples.

### Malondialdehyde (MDA) content

Thiobarbituric acid reactive substances (TBARS)/MDA equivalent production is an index of membrane lipid peroxidation^41^. Since there was similar amount of H_2_O_2_ production in both the control and water-stressed seedlings (after 24 h of greening), we did not observe any significant increase in MDA content either. After 72 h of greening under water-stress, the MDA content increased by 50% above the control value, as expected (Fig. 6B).

### Differentially expressed proteins

**Figure 7:**
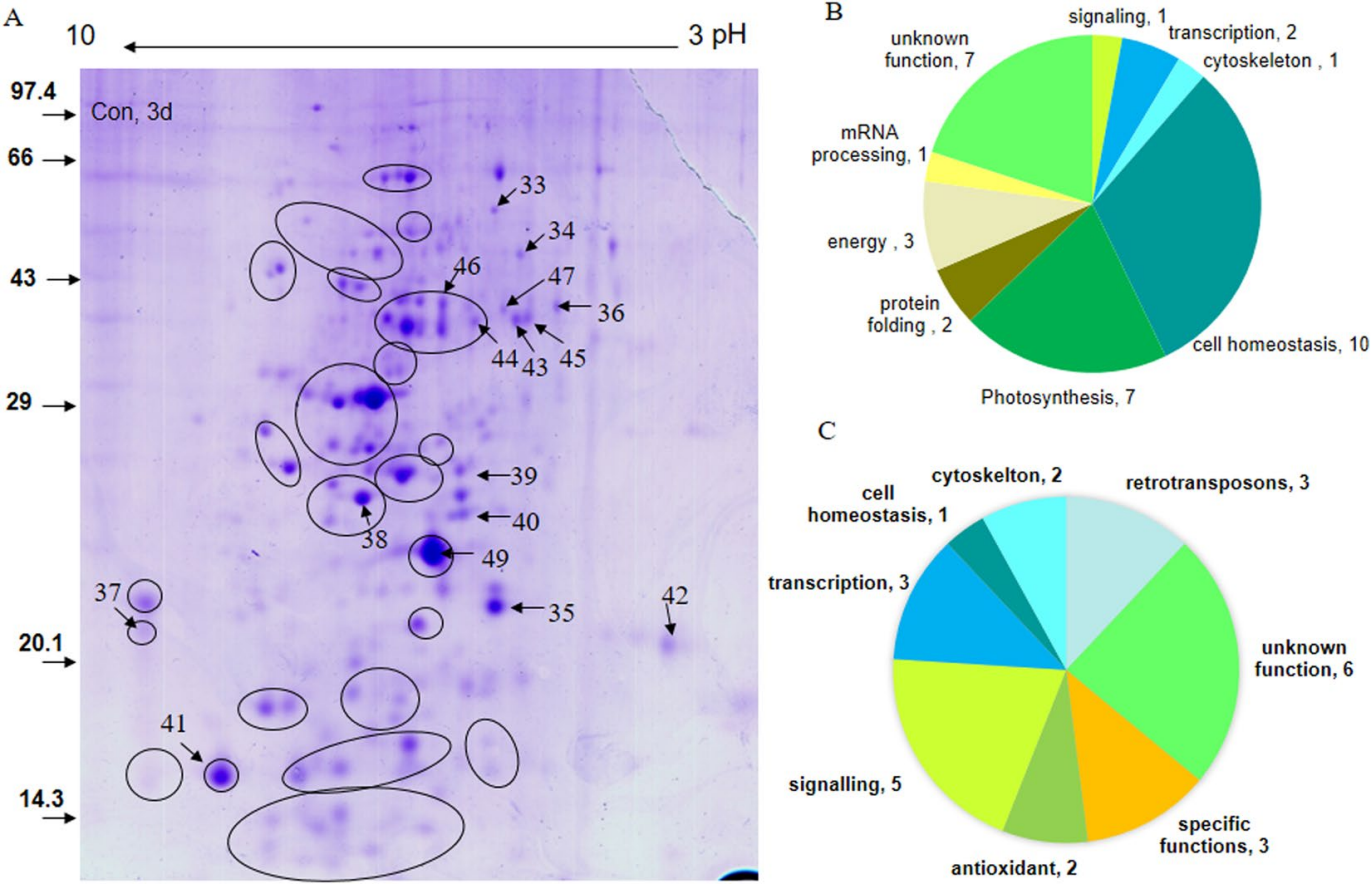
Proteomics of PEG fractionated soluble and peripheral thylakoid proteins of rice leaves after 72 h of water-stress treatment in light. Five-day old etiolated seedlings were subject to water-stress as in Fig. 1. The 2D gel of control and treated samples were done several times till a consistent pattern was obtained. (**A**) Representative 2-D gel of PEG fractionated soluble proteins of control rice seedlings, (**B**) functional category distribution of 34 down-regulated and (**C**) 25 up-regulated differentially expressed proteins based on actual number of identified proteins. Soluble proteome was isolated and PEG fractionated in two steps; first upto 10% and later upto 20% to remove abundant proteins e.g. Rubisco. Protein (800 µg) from supernatant was treated with G-Biosciences Perfect Focus kit to remove impurities; the obtained pellet was air dried and dissolved in rehydration buffer. After rehydration, isoelectric focusing of proteins was done on a 17 cm IP strip overnight; strip was equilibrated and second dimension was run on a 12.5% SDS-PAGE. 2-D gel was stained with Coomassie Brilliant Blue (CBB), Imaged with UMAX PowerLook 2100XL Image Scanner. Spots were detected, and matched with the Image Master-2D Platinum 6.0 software to get differentially expressed proteins. Numbered spots showing down-regulated proteins in the representative gel from control seedlings are mentioned in text and tables starting with CBC3.

Soluble and peripheral membrane proteome was analyzed by 2-D gel electrophoresis and MALDI-TOF/TOF or ESI-MS/MS analysis of spots, after 72 h of greening. Silver-staining as well as colloidal Coomassie Brilliant Blue (CBB)-staining was used to visualize the spots (Fig. 7A). In silver-stained gel, we identified 31 differentially expressed proteins; 10 were up-regulated and 21 were down-regulated. In CBB-stained gels, we further identified 15 up-regulated and 13 down-regulated proteins. In total, 34 down-regulated proteins and 25 up-regulated proteins were identified (Table 1): Silver-stained proteins are named starting with ‘SS’, and CBB-stained spots are named staring with ‘CB’; C3 and W3 denote spots from the ‘control’ and the ‘water-stressed’ samples, respectively, after 72 h/3 d of greening. Gene Annotations, Gene Index (GI) number, locus names, gene names and localization (wherever known or predicted) for identified proteins are provided in the Supplementary Table S1. Out of 59 differentially expressed identified proteins, for 18 Uniprot IDs, no gene models were retrieved from the rice database (http://ricedb.plantenergy.uwa.edu.au/). For the remaining 41 (59–18) Uniprot IDs, 56 corresponding gene-model/loci were retrieved as shown in the Supplementary Table S1. GO enrichment analysis performed using loci given in Supplementary Table S3, is provided in the Supplementary Table S2. Enriched GO-terms (Biological Process) (p < 0.05) were glycolysis, tricarboxlic acid cycle, cellular carbohydrate metabolic process, oxidation-reduction, cysteine biosynthetic process, photosystem II stabilization, carbon fixation, hydrogen peroxide catabolic process, cysteine biosynthetic process from serine, phosphatidyl inositol metabolic process and phosphate metabolic process.

**Table 1:** Differentially expressed proteins in water-stressed rice seedlings categorized into different subgroups according to their function.

Spot No	Protein	Mowse Score	Mr (kD)/pI	Access key	Sequence Coverage/peptides matched	Protein orthologue	Up/down regul -ated
**1. Carbon fixation:**							
SSC3-2	Ribulose bisphosphate carboxylase large chain precursor, putative [Oryza sativa (japonica cultivar−)]	70.5	56.5**/**9.7	gi|108862318	19.3%/10		Down
SSC3-8	Fructose-bisphosphate aldolase	113	41.8**/**6.0	gi|108864048	34.4%/13		Down
SSC3-12	Fructose-bisphosphate aldolase class-I [Oryza sativa Japonica Group]	77.9	39.6**/**7.6	gi|62732954	23.7%/8		Down
SSC3-20	hypothetical protein OsJ_30136 [Oryza sativa Japonica Group]	137	26.3**/**9.7	gi|125606445	60.0%/15	Triose phosphate isomerase, chloroplastic	Down
SSC3-23	hypothetical protein OsI_20474 [Oryza sativa Indica Group]	109	30.5**/**7.7	gi|125552851	43.7%/10	Phosphoglycerate kinase, chloroplastic	Down
SSC3-11	Os01g0501800 [Oryza sativa (japonica cultivar-group)]	143	35.1**/**6.0	gi|115436780	53.8%/15	OEC 33	Down
CBC3-49	PSII Oxygen evolving complex protein 2 precursor, rice	332	26.932**/**8.66	T02B73	44%/8		Down
**2. Unknown/Uncharacterized proteins:**							
SSC3-1	Os04g0620200 [Oryza sativa (japonica cultivar-group)]	45.8	31.6**/**9.1	gi|115460610	24.3%/5	Remorin C-terminal domain protein+ DNA binding	Down
SSC3-6	hypothetical protein LOC_Os11g39500 [Oryza sativa (japonica cultivar-group)]	33.1	13.7**/**9.8	gi|77551932	18.6%/3	Hypothetical protein	Down
SSC3-7	hypothetical protein OsI_32755 [Oryza sativa Indica Group]	34.9	11.5**/**11.1	gi|218184148	49.0%/3	IGR family prot (unknown function)	Down
SSC3-18	Os01g0652000 [Oryza sativa (japonica cultivar-group)]	56.4	11.3**/**10.7	gi|115438911	60.8%/5	Uncharacterized	Down
SSC3-19	unknown protein [Oryza sativa Japonica Group]	44.3	12.7**/**10.4	gi|47900462	28.6%/3	Unknown protein	Down
CBC3-39	hypothetical protein [Oryza sativa Japonica Group]	28.5	12.8/6.7	gi|53791471	51.7%/2	No match	Down
CBC3-40	hypothetical protein [Oryza sativa Japonica Group]	59	9.9/7.8	gi|57899958	46.4%	No match	Down
CBW3-5	hypothetical protein [*Oryza sativa*]	45.2	15.2/11.5	gi|10440615	59.4%/6	No match	Up
CBW3-26	hypothetical protein [*Oryza sativa* Japonica Group]	38.2	16.01/11.6	gi|51534990	30.8%/4	No match	Up
CBW3-27	hypothetical protein OsI_26020 [*Oryza sativa* Indica Group]	43.1	260.7/10.8	gi|218199617	22.8%/8	No match	Up
CBW3-29	Os12g0623600 [Oryza sativa (japonica cultivar-group)]	34.0	32.36/7.8	gi|115489646	18.8%/3	Unknown function RmlC_Cupin domain, DUF1637 family	Up
CBW3-31	hypothetical protein OsI_02030 [Oryza sativa Indica Group]	46.4	12.58/9.9	gi|125526034	37.9%/4	No match	Up
CBW3-32	hypothetical protein [Oryza sativa Japonica Group]	17.54/31.4	12.4	gi|50251877	28.2%/32	No match	Up
**3. Cell Homeostasis:**							
SSC3-4	hypothetical protein OsJ_22283 [Oryza sativa Japonica Group]	51.9	18.6**/**10.1	gi|222636049	20.9%/4	Thioredoxin O; Mitochondrial, OsTrxO1	Down
SSC3-9	Os01g0978100 [Oryza sativa (japonica cultivar−)]	138	42.1**/**6.3	gi|115442595	48.7%/16	Putative plastidic cysteine synthase1	Down
SSC3-13	hypothetical protein LOC_Os03g32290 [Oryza sativa (japonica cultivar-group)]; predicted mitochondrial with TargetP	42.4	11.7**/**5.4	gi|108709024	33.0%/3	Hypothetical protein; low similarity (~51% positives) to Glycerol 3-phosphate dehydrogense	Down
SSC3-14	aminotransferase-like [Oryza sativa Japonica Group]	54.9	20.0**/**4.8	gi|50725174	37.3%/4		Down
SSC3-16	hypothetical protein OsJ_04099 [Oryza sativa Japonica Group]	51.9	80.6**/**10.1	gi|222619550	14.6%/6	Chloroplastic, anion transporter 3; have LRR and receptor kinase like domain; Alternate phosphate transporter 4	Down
CBC3-43	Putative malate dehydrogenaseOryza sativa (japonica cultivar -group)	306	35.414**/**8.22	Q6F361_ORYSA	30%/12		Down
CBC3-44	Putative malate dehydrogenase Oryza sativa (japonica cultivar -group)	215	35.439**/**8.74	Q94JA2_ORYSA	22%/7		Down
CBC3-45	Putative malate dehydrogenase Oryza sativa (japonica cultivar -group)	103	35.414/8.22	Q6F361_ORYSA	12%/4		Down
CBC3-46	Malate dehydrogenase cytoplasmic, Oryza sativa (japonica cultivar)	221	35.415**/**5.75	MDHC_ORYSA	34%/17		Down
CBC3-47	Putative malate dehydrogenase mitochondrial Oryza sativa (japonica cultivar -group)	124	35.439**/**8.74	Q94JA2_ORYSA	12%/5		Down
CBW3 -24	Os04g0174100 [*Oryza sativa* (japonica cultivar-group)]	36.9	27.7/9.0	gi|115457106	21.7%/4	(CYP family, fragment) or tyrosine N-monooxygenase	Up
**4. Retrotransposons:**							
SS W3-7	putative gag-pol polyprotein [Oryza sativa (japonica cultivar-group)]	53.9	133.1**/**6.9	gi|31126757	9.5%/8		Up
SS W3-8	OSIGBa0146I21.3 [Oryza sativa (indica cultivar-group)]	54	79.35**/**9.8	gi|116309588	9.1%/7	retrotransposon protein, putative, Ty1-copia subcla	Up
SS W3-11	Hypothetical protein P0701E03.31.- Oryza sativa (japonica cultivar-group).	18.3	6.77**/**4.7	Q69YD6_ORYSA	10.6%/1	plant disease resistance polyprotein-like	up
**5. Transcription factors and DNA/RNA binding proteins:**							
CBC3-35	Putative glycine rich protein 2 Oryza sativa-japonica; have four CCHC Zn fingers	49	22.709/6.64	Q6YUR8_ORYSA; BAD07599	5%/1	Contain S1-like CSD; cold shock domain protein 1	down
CBC3-41	Os08g0518200 [Oryza sativa (japonica cultivar-group)]	66.7	10.2/72.15	gi|115477308	25.3%/12	Mito transcription termination factor family -related protein	down
SS W3-14	Zinc finger CCCH domain-containing protein 31 OS = O.sativa sub. japonica GN = Os04g0665700 PE = 2 SV = 1	51.3	32.338**/**10.4	C3H31_ORYSJ	29.4%/5		up
CBW3-4	hypothetical protein OsI_09486 [*O. sativa* Indica]; C2H2 Zn-finger	45.7	20.5/4.9	gi|125541662	13.8%/5	Low Similarity (44%) to *Setaria* Zn-finger prot 7-like	up
CBW3-25	hypothetical protein [*Oryza sativa* Japonica Group]	32.6	11.8/12.6	gi|53793156	46.7%/4	RNA binding glycine-rich protein like	up
**6. Signaling:**							
SSC3-22	Os06g0146100 [Oryza sativa (japonica cultivar-group)]	47.3	118.9**/**9.2	gi|115466384	9.5%/9	NBS-LRR disease resistant protein	down
SS W3-12	Hypothetical protein OSJNBb0005A04.22.- Oryza sativa (japonica cultivar).	59.8	51.87**/**11.2	Q6EQ30_ORYSA	25.9%/6	calcineurin-like phosphoesterase-like	up
SS W3-13	hypothetical protein OsI_14142 [Oryza sativa Indica Group]	65.5	49.62**/**9.5	gi|218194019	30.0%/12	Putative receptor kinase (tyrosine)	up
SS W3-15	Os09g0483100 [Oryza sativa (japonica cultivar-group)]	59.3	15.076**/**4.2	gi|115479825	65.2%/5	Ca^+2^ binding EF-hand 5 motif prot	up
CBW3-1	hypothetical protein - OSJNBa0016I09.17.- *Oryza sativa* (japonica cultivar)	49.5	30.4/10.7	Q5QNA8_ORYSA	26.4%/5	Similar to receptor like kinase	up
CBW3-10	Phosphatidylinositol-4-phosphate 5-kinase 4, putative, expressed [*Oryza sativa* (japonica cultivar)]	53.5	84.93/9.7	gi|108708236	13.5%/8		up
**7. Energy balance:**							
SSC3-15	Os02g0750100 [Oryza sativa (japonica cultivar-group)]	57.2	26.2**/**4.8	gi|115448701	23.5%/6	H+ transporting ATP synthase, δ chain	down
SSC3-21	putative inorganic pyrophosphatase [Oryza sativa Japonica Group]	155	31.8**/**5.7	gi|46805452	42.3%/14		down
CBC3-34	AP003518 NID: Oryza sativa- japonica cultivar	58	41.315/6.68	BAD37348	6%/3	Formate dehydrogenase, mitochondrial precursor	down
**8. Protein folding/de-folding and degradation:**							
SSC3-3	putative Lon protease [Oryza sativa Japonica Group]	33.1	96.9/7.0	gi|50725794	6.9%/4		down
CBC3-42	Putative peptidylprolyl isomerase Oryza sativa (japonica cultivar -group)	140	26.574/9.37	Q75M32_ORYSA	24%/5		down
**9. Antioxidant proteins:**							
CBW3-28	Os03g0285700 [*Oryza sativa* (japonica cultivar-group)]	66.4	27.02/5.3	gi|115452337	54.8%/10	APx1	up
CBW3-30	Os05g0116100 [Oryza sativa (japonica cultivar-group)]	141	23.44/5.8	gi|115461741	41.8%/9	GST_C family, DHAR	up
**10. Cell shape/cytoskeleton protein:**							
SSW3-6	Os04g0538800 [Oryza sativa (japonica cultivar-group)]	71.5	106.4**/**6.6	gi|115459666	12.3%/8	Similar to Kinesin heavy chain (fragment)	up
SSC3-5	hypothetical protein OsI_28608 [Oryza sativa Indica Group]	39.9	57.6**/**0.0	gi|125560931	0.0%/3	Similar to Transducin/WD-40 repeat protein-like	down
CBW3-3	Hypothetical protein.-*Oryza sativa* (japonica cultivar-group). Os12g0465000	28	14.1/10.2	Q2QRD6_ORYSA	22.0%/2	Similar (68% positives) to Saccharum hybrid R570, A0A059Q350 Acetylglucosaminyl transferase family protein	up
**11. mRNA processing:**							
CBC3-33	Hypothetical Protein OJ1611_C08.15.-Oryza sativa (japonica cultivar -group)	39	132.188/5.57	Q6ZHF3_ORYSA	1%/1	UPF0614 C14orf102-like protein; have HAT and NRDE-2 domains	down
**12. Proteins with specific functions:**							
SSW3-9	IAA-amino acid hydrolase ILR1-like 3 O.sativa sub japon ica GN = ILL3 PE = 2 SV = 1	47.8	44.07**/**5.4	ILL3_ORYSJ	23.0%/5		up
SSW3-10	IAA-amino acid hydrolase ILR1-like 3 OS = Oryza sativa subsp. japonica	47.4	44.07**/**5.4	ILL3_ORYSJ	17.0%/6		up
CBW3-2	Salt stress-induced protein (Salt protein) (Protein mannose-binding lectin).- *Oryza sativa*	72	15.03/4.9	SALT_ORYSA	45.8%/5		up

^a^Spot No. generated by UMAX PowerLook 2100XL Image Scanner (v 6.0, GE Healthcare); Numbered spots correspond to the identified proteins,.refer the spots shown on the representative image in Fig. 7. ^b^Accession number from NCBI database. SC, sequence coverage; Mr, theoretical Molecular weight; pI, theoretical Isoelectric point; Fold change refers to increased (Up) or decreased levels (down) of protein content from control to water-stressed seedlings. Fold change ± 2 was used as threshold for protein identification. Orthologues were obtained by using Uniprot protein sequence and BLAST.

Category-wise distribution of down-regulated and up-regulated identified proteins is presented in Fig. 7A and B respectively. Differentially-regulated proteins were functionally distributed into 12 groups (Table 1).

Down-regulated proteins (Table 1, and Supplementary Table S1) were categorized into 9 groups (Fig. 7A), namely: (**i**) photosynthesis (“light reaction”; OEC23, OEC33 and carbon fixation; Rubisco LSU, FBA, PGK, TPI); (**ii**) protein folding and degradation (PPIase and Lon protease); (**iii**) energy balance (putative ATP synthase δ subunit; inorganic pyrophosphatase and mitochondrial formate dehydrogenase precursor); (**iv**) transcription/RNA stabilization (mTERF and CSD protein); (**v**) cytoskeleton organization (similar to WD40 domain containing and kinesin like); (**vi**) cell homeostasis (Cyt P450 protein, TRX domain containing protein, cysteine synthase 1, glycerol 3-phosphate dehydrogenase (GPDH), malate dehydrogenase (MDH), aminotransferase like and anion transporting protein); **(vii**) mRNA processing (UPF0614 protein C14orf102); (**viii**) signaling; and (**ix**) unknown/uncharacterized proteins.

The proteins that were up-regulated (Table 1 and Supplementary Table S1) in water-stressed seedlings were distributed into 8 categories (Fig. 7B), which are: (**i**) signaling (receptor like kinase, putative receptor kinase, phosphatidyl inositol 4-phosphate 5-kinase 4 (PIP5K4), calcineurin-like phosphoesterase like, Ca^2+^ binding EF hand-5 domain containing); (**ii**) transcription/RNA stabilization (C_2_H_2_ Zn-Finger, C_3_H Zn finger, RNA binding protein); (**iii**) retrotransposons (Ty-1 copia type; putative gag pol polymerase and plant disease resistance polyprotein-like retrotransposon); (**iv**) antioxidant defense mechanism (ascorbate peroxidase (APx1), and dehydro ascorbate reductase (DHAR)); (**v**) cytoskeleton organization (Kinesin heavy chain like and Glucosyl-galactosyl transferase); (**vi**) cell homeostasis (Cyt P450); (**vii**) specific functions (SalT; IAA amino acid hydrolase ILR1-like 3); and (**viii**) uncharacterized proteins.

## Discussion

During photomorphogenesis, Chl, proteins, and lipids are synthesized and assembled to form the functional photosynthetic apparatus. Earlier, Dalal and Tripathy^33^ showed that water-stress during transition from skotomorphogenesis to photomorphogenesis led to a 42% reduction in Chl content, due to down-regulation of expression of genes and proteins involved in Chl biosynthesis^33^. This must be, at least partly, responsible for the lower Fo and Fm (Fig. 1A), as well as the lower Chl a fluorescence in water-stressed rice seedlings (Fig. 4A). Shibata shift (blue shift of absorption maximum of chlorophyllide from 684 nm (aggregated form) to 672 nm (disaggregated form), discovered by K. Shibata^42^ that takes place within a few minutes of light exposure of etiolated seedlings was also impaired in stressed seedlings^33^. In higher plants, both Chl *a* and Chl *b* are bound to light-harvesting pigment-protein complexes, LHCs. The availability of Chl *b* is essential for the assembly and functioning of most LHC proteins^43^. Binding of Chls to the LHC proteins stabilizes the latter in the thylakoid membranes^44^. Further, ROS produced in chloroplasts of water-stressed plants could down-regulate the expression of genes involved in Chl biosynthesis and photosynthesis via retrograde signaling^45,46^. In water-stressed rice seedlings, there was not only a downregulation of Chl biosynthesis, but also a reduction in pigment-protein complexes; this, obviously, led to lower Chl *a* fluorescence (Fig. 2). Moreover, biosynthesis of PSII reaction center protein D1 and its repair were also inhibited by H_2_O_2_, and other photosynthetic inhibitors and uncouplers^47,48^. In water-stressed developing rice seedlings components of light-harvesting complexes of both PSII and PSI i.e., Lhcb1, Lhcb2, Lhca1 and Lhca4 decreased (Fig. 4). Lack of Chl *b* in *ch1* mutant of *Arabidopsis* resulted in the absence of accumulation of LHCs, even though they had normal mRNA expression for the LHCs^49^. Conversely, an increase of Chl *b* and total Chl content in tobacco upregulated gene expression and protein abundance of different pigment-protein complexes^50^.

In contrast to our present observation in developing rice seedlings, water-stress imposed on four-week-old well developed green wheat plants had no effect on the leaf chlorophyll content, and on the abundance of ATP synthase, PSII, PSI, and light-harvesting complexes^51^. However, in these plants, thylakoid membranes and other proteins were oxidized, due to the generation of ROS as a consequence of excess light absorption by the LHCs^51^. Decreased LHCII is an essential protection mechanism that allows plants to survive under unfavourable conditions^52^. Our results demonstrate that germinating seedlings protect themselves from water-stress-induced oxidative stress and photo-damage by downsizing their light-harvesting antenna and photosynthetic reaction centers (Figs 4 and 6).

The decreased Fo and Fm (Fig. 1A) in water-stressed rice seedlings is most likely due to a reduced Chl content^33^, and/or stress-induced alteration in xanthophyll-cycle dependent non-radiative energy dissipation^53^. Alternatively, low fluorescence may be related to a state 2 induced during the water-stress treatment. A significant decrease in Fv/Fm, which is a proxy of PSII photochemistry efficiency^37^, in water-stressed developing seedlings (Fig. 1A), might have resulted from the down-regulation of synthesis and assembly of PSII reaction centers. This would have reduced not only the concentration of PSII reaction centers, but also of the oxygen evolving complex proteins, such as OEC33 (PsbO), OEC23 (PsbP) and OEC 16 (PsbQ) (Fig. 4). The observed changes in Chl *a* fluorescence spectra (Fig. 2) are in agreement with this observation. Furthermore, the “operating” quantum yield of PSII (ϕPSII) during actinic illumination also sharply declined in water-stressed developing seedlings (Fig. 1B), OEC33 is important for stabilization of Mn and Cl co-factors in water oxidizing complexes^54^; its reduction *via* RNAi results in a decrease in quantum yield of photosynthesis^55^. Beside providing appropriate amounts of calcium and chloride ions for water splitting reactions, OEC23 and OEC16 play an additional role in the assembly of PSII supercomplexes, and their decreased abundance was clearly correlated with reduced PSII-dependent O_2_ evolution^54^; therefore, down-regulation of OEC proteins also explains the impaired PSII-dependent water oxidation and oxygen evolution that we found in thylakoid membranes isolated from water-stressed developing seedlings (Fig. 3).

Higher NPQ in water-stressed seedlings compared to the controls at all measured PAR values, especially at low actinic light intensities (Fig. 1C**)** suggest an adaptive response of developing seedlings for increased dissipation of absorbed energy as heat to protect their thylakoid membranes from photodamage^29^. Down-regulation of δ-subunit of ATP synthase was observed in our proteome profile of water-stressed seedlings (Table 1). Impaired ATP synthase would have resulted in reduced transport of protons across the thylakoid membrane; the lowered dissipation of ΔpH and increased acidification of the lumen^56^ could lead to higher NPQ in (water) stressed seedlings even at low light intensities.

In water-stressed samples, reduced amount of Chl, LHCs and reaction center proteins result in reduced light absorption, and decreased amount of oxygen evolving complex proteins result in reduced utilization of the absorbed energy. Consequently, reduction of PSII activity was almost similar (nearly 50%) under limiting as well as under saturating light intensities. In the same vein, the downsizing of the light-harvesting Chl-binding proteins of PSI i.e., LHCI (Lhca1 and Lhca4) and PSI core complex protein subunits III (PsaF), V (PsaK) and VI (PsaH) in water-stressed seedlings, resulted in diminished light absorption as well as light utilization by PSI, as was evident from the reduction of PSI reaction by 30% at limiting and higher light intensities (Fig. 3E). Relatively lower reduction of PSI activity than that of PSII (Fig. 3) suggests that development of PSI of developing rice seedlings is more tolerant to water-stress. The Eadie plot^31,40^ for control and treated seedlings revealed a marked drop in the quantum efficiency of PSI and PSII reactions and a substantial reduction of Vmax in stressed developing seedlings (Fig. 3); these suggest a coordinated developmental down-regulation of light-harvesting and reaction center proteins of PSI as well as PSII in response to osmotic stress^31,40^.

Room temperature fluorescence emission spectrum of thylakoid membranes had a peak at 684 nm due to PSII, when they were excited at 440 nm (Fig. 2A). Water-stressed samples had a reduced fluorescence emission, probably due to a block in electron donation from water side of PSII; decreased abundance of oxygen evolving complex proteins (33 kDa, 23 kDa and 18 kDa) supports this hypothesis. Gross perturbation of structural organization of thylakoid membranes usually induces differences in fluorescence spectra at low temperature (77 K); these spectra usually have emission peaks at 685 nm (F_685_), at 695 nm (F_695_), which mostly originates from PSII CP43 and CP47 respectively^38^, and a F_735-740_ peak that originates mostly from PSI^57^. If a part of LHCI antenna is removed from PSI by detergent treatment, the inner antenna of the PSI reaction center fluoresces at 722 nm^58^, while isolated LHCI complexes fluoresce around 735–740 nm; this is consistent with the assignment by Briantais *et al*.^59^: the inner PSI antenna for F_722_ and for LHC I for F_735–740_^59^. A shift to F_738_ from F_740_ in our water-stressed samples are due to a partial loss of components of LHCI.

In the absence of Mg^2+^, the F_686_/F_740_ ratio in thylakoid membranes isolated from control seedlings was ~1.54 (Fig. 2B). In experiments by others, LHCII has been shown to migrate closer to PSII^60^ in presence of  Mg^+2^, leading to stacking of thylakoids into granum^61^ (see a review on role of ions by Kaňa and Govindjee)^62^. This results in efficient energy transfer from LHCII to the reaction center of PSII, and consequently, an increase in PSII fluorescence at 686 nm and, thus, higher F_686_/F_740_ [*see Results*] ratio (an increase of F_686_/F_740_ ratio to 1.73 in the control samples with Mg^2+^, compared to 1.54 without Mg^2+^ (Fig. 2B)). In water-stressed seedlings, Mg^+2^-induced increase in F_686_/F_740_ was almost similar to control. Although a reduced grana stacking was observed in electron micrographs of the plastids of water-stressed seedlings, they retained the ability of Mg^2+^-induced migration of LHCPII to PSII core complex.

As revealed from our proteomics studies, water-stressed rice seedlings downsized their carbon reduction cycle enzymes i.e., Rubisco LSU, fructose bisphosphate aldolase (FBA), triosephosphate isomerase (TPI) and phosphoglycerate kinase (3PGK) (Table 1). In contrast, in water-stressed well-developed plants, Rubisco content was often not affected, although its initial and total activity is known to decrease due to blocking of catalytic sites by 2-carboxyarabinitol-1-phosphate and other sugar phosphates^63^. Sugar phosphates can be removed from Rubisco by ATP-dependent enzyme Rubisco activase^17^, leading to its reactivation, hence underlining the role of Rubisco activase and ATP production in regulating carbon assimilation under drought. Drought-induced stomatal closure, and consequent decreased carboxylation, also result in down-regulation of protein abundance of Calvin-Benson cycle enzymes^13–16^.

Down-regulation of peptidyl prolyl isomerase (PPIase), in our water-stressed rice seedlings, suggest that there was a decrease in the ability to repair photosynthetic proteins, which, in turn, results in reduced PSI and PSII activities.

In mature plants, only minimal amounts of Chl and proteins are synthesized, mostly to replace those  photodestroyed in PSI and PSII and LHCs, due to light. Therefore, it appears that mature plants do not downsize the components of the photosynthetic apparatus in response to drought. Several studies have demonstrated that abiotic stresses, including water-stress, cause ROS production due to an over-reduction of photosynthetic electron transport chain^25,29,64,65^. Abiotic stresses also impair reaction centers of well-developed seedlings or mature plants, leading to reduced transfer of absorbed light energy from light harvesting complexes to photodamaged reaction centers and consequently generation of higher amounts of singlet oxygen via photosensitization reactions of Chl^27,28,31^. Well developed rice plants treated for 48 h with 30% PEG 6000, a concentration similar to that used in our current experimental protocol, accumulated 2.5–4 fold higher H_2_O_2_, which impaired Rubisco activity and promoted stomatal closure^25^. However, we showed that germinating seedlings protect themselves by downsizing their light-harvesting antenna and photosynthetic reaction centers to reduce ROS production (Figs 4 and 6). In addition, we found that antioxidative enzymes i.e., ascorbate peroxidase 1 (APx1) and dehydroascorbate reductase (DHAR), were up-regulated in developing stressed seedlings (Table 1) to neutralize comparatively smaller amount of ROS generated. Therefore, we did not observe any increase of H_2_O_2_ in developing seedlings subjected to water-stress for 24 h, and there was only a small increase (23%) after 72 h. Consequently, membrane lipid peroxidation, monitored as MDA production did not increase after 24 h of stress treatment, but it increased by 50% only after 72 h.

In conclusion, we can clearly state that unlike mature plants, seedlings exposed to water-stress during early photomorphogenesis, protect themselves from photo-oxidative stress by downsizing their photosynthetic apparatus. Therefore, we recommend that attempts should be made to down-regulate the light absorption during stress condition by reducing Chl biosynthesis. This can be achieved by either using molecular marker assisted plant breeding methods or by using transgenic RNAi approaches, silencing the expression of gene encoding for glutamyl-tRNA reductase or other early enzymes responsible for the synthesis of 5-aminolevulinic acid, a precursor of Chl biosynthesis. The RNAi expression could be modulated by promoters that sense increased ROS (e.g., H_2_O_2_) produced during stress conditions.

## Materials and Methods

### Plants growth conditions and stress treatment

Seeds of drought-sensitive rice (*Oryza sativa*) cultivar Pusa Basmati-1 (PB-1) were obtained from the Indian Agricultural Research Institute, New Delhi, India. Five-day old etiolated seedlings, grown at 25 °C, were treated with half-strength MS^66^ solution, or half-strength MS solution +40 mM PEG (−0.73 MPa) or 50 mM PEG 6000 (−1.06 MPa; Merck, Kenilworth, NJ)^35^, 16 h prior to their transfer to continuous cool-white fluorescent (plus incandescent) light of 100 µmol photons m^−2^ s^−1^; low light intensity was used to avoid photodamage of the seedlings during greening. The seedlings were maintained for 3 days at 28 °C and at 75 % relative humidity in a Conviron plant growth chamber.

### Polarographic measurement of photosynthetic electron transport

Approximately 5 g of leaves were homogenized in 40 ml of isolation buffer containing 0.4 M sorbitol, 0.05 M Hepes/KOH (pH 7.3), 1mM MgCl_2_, and 1mM EDTA at 4 °C, under green safe light (28 Chakraborty and Tripathy, 1992). The homogenate was passed through 8 layers of cheese cloth and 1 layer of Mira cloth, and centrifuged at 5,000 rpm for 7 min. The pellet was suspended in a buffer solution containing 0.4 M sorbitol, 0.05 M Tris (pH 7.6), 1 mM MgCl_2_ and 1 mM EDTA; Chl concentration was estimated according to Porra *et al*.^67^.

Photosynthetic electron transport was measured at 25 ± 1 °C with a Clark-type oxygen electrode (Hansatech, Kings Lynn, UK), by using 1 ml suspension of thylakoid membranes containing 20 µg Chl ml^−1^; illumination was with white-light from a tungsten source of 1,500 μmol photons m^−2^ s^−1^. The whole electron transport chain, from H_2_O to methylviologen (MV) (1 mM), was monitored as O_2_ uptake^68,69^. PSII activity was monitored as O_2_ evolution resulting from electron transport from H_2_O to p-phenylenediamine (PD) (0.5 mM). The partial electron transport through PSI was measured as oxygen consumption. Ascorbate (1 mM)/DCIP (0.2 mM) couple was used as electron donor to PSI, and MV (1 mM) as electron acceptor^68,69^; the electron flow from PSII was blocked by 3-(3, 4-dichlorophenyl) 1,1-dimethyl urea (DCMU) (20 μM), and 1 mM sodiumazide was used as an inhibitor of catalase.

Light saturation curves were measured at several light intensities obtained by using neutral density filters (Balzers, Neugrüt, Lichtenstein).

### Transmission electron microscopy

Leaves (in triplicate) were fixed with 2.5% glutaraldehyde^70^ and 1% OsO_4_ for 2–4 h, and then washed, dehydrated with acetone, cleared with epoxy propane/xylene, and infiltrated with resin containing araldite and toluene (up to 75% araldite). After embedding, araldite was polymerized at 50 °C, and then at 60 °C. Ultra-thin sections were cut, stained in saturated uranyl acetate in 50% ethanol, washed in 50% ethanol and water, and viewed in a Transmission Electron Microscope (TEM) (Phillips-CM-10, Eindhoven, Netherlands).

### Chlorophyll a fluorescence measurements

Chl *a* fluorescence signal is a highly sensitive signature of diverse aspects of photosynthesis, and thus, it is widely used in photosynthesis research^38,71^ (see chapters in Papageorgiou and Govindjee)^72^.

#### Fluorescence emission spectra of isolated thylakoids

Room temperature (RT; 298 K) and low temperature (77 K) fluorescence emission spectra of isolated thylakoids were recorded with a SLM-AMINCO-8000 spectrofluorometer; for 77 K-spectra, the excitation and emission slit widths were 4 nm, while for RT-spectra, the excitation and emission slit widths were 8 nm and 4 nm respectively. Rhodamine-B was used in the reference channel as quantum counter. A tetraphenylbutadiene block was used to adjust the voltage in sample, as well as in reference channels to 20000 counts s^−1^ at excitation and emission wavelengths of 348 nm and 422 nm, respectively. RT-spectra were corrected for the instrument response.

#### Thylakoid isolation

Leaves from control and water-stressed seedlings were grounded in Hepes-NaOH buffer, pH 7.6 (20 mM Hepes, 10 mM NaCl, 0.4 M sucrose, 0.5% BSA) and then centrifuged at 5,000 rpm for 7 min; pellet was suspended in 5 mM Hepes-NaOH buffer (pH 7.5) containing 0 or 4 mM MgCl_2_. Thylakoids equivalent to 3 µg Chl ml^−1^ were used for RT- and 77K-spectra (25% glycerol) at excitation wavelength of 440 nm and emission wavelength range of 620–750 nm and of 620–780 nm, respectively^31^. Each experiment was performed three times.

#### Chl a fluorescence induction curves

Chl *a* fluorescence induction curves at 25 °C were recorded on attached leaves, with a portable PAM-2100 fluorometer (Walz, Effeltrich, Germany); all measurements were repeated 3 times. The initial (Fo) and maximum (Fm) fluorescence, was measured on seedlings that were dark-adapted for 20 min; the measuring light was red (650 nm), and of very low intensity (<0.1 μmol photons m^−2^ s^−1^); its frequency was 0.6 KHz. Then, a 0.8 s saturation light pulse of approximately 8,000 μ mol photons m^−2^ s^−1^ was applied to measure the maximum fluorescence, Fm. The Fo and the Fm values were used to calculate the ratio Fv/Fm = (Fm − Fo)/Fm, where Fv is the maximum variable fluorescence; this ratio represents the maximum quantum yield of PSII photochemistry. Light response curves were obtained by measuring fluorescence as a function of increasing actinic light intensity (4 to 270 μmol photons m^−2^ s^−1^; wavelength: 665 nm). The quantum yield of PSII (ϕPSII) was calculated as (Fm′ − Ft)/Fm′, where Fm′ is the maximum fluorescence at steady state in pre-illuminated samples, and Ft is fluorescence immediately before the application of the saturation pulse^37^. Non-photochemical quenching (NPQ) was calculated as (Fm − Fm′)/Fm′.

### Thylakoid isolation and western blotting

20 μg of plastid protein^28^, estimated as described by Bradford^73^, was separated on SDS-PAGE three times for each photosynthetic target protein, transferred to nitrocellulose (NC) membranes and blocked with BSA solution. Subsequently, blots were incubated with polyclonal and heterologous primary antibodies (Supplementary Table S4) and alkaline phosphatase-conjugated antibodies and developed for color. Image J (NIH, USA) was used for quantification.

### Estimation of malondialdehyde (MDA)

200 mg fresh tissue was homogenized in 20 % (w/v) trichloroacetic acid (TCA) alone (−TBA (Thiobarbituric acid)) or 20 % TCA (w/v) plus 0.25 % TBA (+TBA)^41^. Samples were vortexed, heated at 95 °C in a water bath for 25 min, cooled, and centrifuged at 12,000 rpm for 10 min. Absorbance was read at 440, 532, and 600 nm. MDA equivalent was calculated as follows: A = (Abs 532_+TBA_) − (Abs 600_+TBA_) − (Abs 532_−TBA_) − (Abs 600_−TBA_)); B = (Abs 440_+TBA_−Abs 600_+TBA_) 0.057; and finally as: (A − B/1,57,000) 10^6^ (in nmol ml^−1^).

### Determination of hydrogen peroxide

Leaf tissue (200 mg) was homogenized in 2 ml of 0.1 % (w/v) TCA solution on ice^74^ and centrifuged at 12,000g for 15 min. 0.4 ml of the supernatant was added to 0.4 ml of 10 mM potassium phosphate buffer (pH 7.0) and 0.8 ml of 1 M KI; the absorbance was measured at 390 nm. The leaf H_2_O_2_ content was measured using standard calibration curve of H_2_O_2_ (Thermo Fisher Scientific, MA).

### Statistical analysis

All measurements were subjected to analysis of variance (ANOVA) to check for significance level of differences (defined as p < 0.05). Data is shown as mean ± standard deviation (SD).

### 2-Dimensional gel electrophoresis

#### Isolation of soluble and loosely bound thylakoid proteins

After 72 h of greening, two grams of tissue was homogenized in 10 ml of ice-cold Mg/NP-40 extraction buffer containing 0.5 M Tris-HCl, pH 8.3, 2% v/v NP-40, 20 mM MgCl_2_, 2% v/v β-mercaptoethanol, 1 mM phenylmethylsulfonyl fluoride and 1% w/v polyvinylpolypyrrolidone^75^; this was repeated several times until a consistent pattern was obtained. After centrifugation at 12,000 *g* for 15 min at 4 °C, 50% (w/v) PEG-4000 stock solution was added to the supernatant to a final concentration of 10% PEG. The solution was incubated in ice and centrifuged at 1,500 g for 15 min each. The supernatant was adjusted to 15% (Silver-stained) or 20% (CBB-stained) PEG by adding 50% (w/v) PEG-4000 stock, incubated and centrifuged as described above. For silver- and CBB-stained gels, 150 µg and 800 µg of protein from resulting supernatant was treated with PERFECT-FOCUS^TM^ kit from Geno Biosciences (St. Louis, MO); the pellet, thus obtained, was washed and air dried.

#### Isoelectric focusing and 2-D gel-electrophoresis

Air dried pellet was dissolved in rehydration buffer containing 8 M urea, 3% CHAPS, 20 mM DTT, 1 mM PMSF, and 2% ampholyte (GE-Healthcare, Buckinghamshire, UK). Protein extract was loaded on a 17 cm IPG strip of pH 3–10 (4–7 in case of silver-stained gels) (Bio Rad, CA). Rehydration was followed by electrophoresis at 250 V for 0.5 h, 10,000 V for 3.5 h, and 40,000 VH at 10,000 V.

After equilibration of the strips, proteins were separated on 12.5% SDS-PAGE. Gels were scanned with UMAX PowerLook-2100XL Image Scanner (GE-Healthcare). Spots were detected, matched, and edited with Image Master-2D Platinum 6.0 software (GE-Healthcare).

### Mass spectrometry and database search

Protein spots showing two fold or higher difference (% vol) were excised, destained, digested and extracted^76^. For one set of spots, matrix-assisted laser desorption ionization (MALDI) with Bruker Autoflex II TOF/TOF mass spectrometer (Bruker Daltonics, Billerica, MI) was used^76^. The peptide mass data were searched against NCBInr (non-redundant) *Oryza sativa*/green plant database with the Mascot search engine (Matrix Science Ltd., UK). Two missed cleavage sites were allowed, cysteine was carbamidomethylated and methionine was allowed to be partially oxidized.

Other sets of proteins were detected with LC-MS/MS^12^. MS analysis was performed on Applied Biosystems 4000-Q-TRAP-system (Foster City, CA), equipped with Agilent 1200-NanoLC system (Santa Clara, CA). Proteins were excised manually, destained, digested and extracted. LC separation of peptides was performed on ZORBAX 300SB-C18 silica column, increasing the gradient from 3 to 97 % acetonitrile in 0.1% formic acid. The first MS detected ions were at scan range of 400–1600 m/z. Fragmentation spectra were submitted by Sequest algorithm to BioWorks (v3.3.1; Thermo Scientific) for searching a composite database of proteins using mascot (http://www.matrixscience.com/). Search settings applied were: trypsin as cleaving enzyme, maximally two missed cleavages, peptide mass tolerance 15 ppm, fragment ion tolerance 0.5 Da, and no differential post-translational modifications were allowed per peptide.

UNIPROT and University of Western Australia rice database (http://ricedb.plantenergy.uwa.edu.au/)^77^ were used for gene annotations of identified proteins. Gene ontology (GO) enrichment analysis was performed with ricearray (http://www.ricearray.org/analysis/go_enrichment.php) using default parameters.

### Data availability

Most of the data generated or analysed during this study are included in this published article (and its supplementary information files). Any other remaining necessary data is available from the corresponding author upon reasonable request.

## Electronic supplementary material

Supplimentary Information

Supplementary Tables
