# Supplementary material for: Water-stress induced downsizing of light-harvesting antenna complex protects developing rice seedlings from photo-oxidative damage
Source: Sci Rep. 2018 Apr 13;8:5955. doi: 10.1038/s41598-017-14419-4 (PMC5899091; doi:10.1038/s41598-017-14419-4)
Supplement: Supplementary file 1 — Supplimentary Information [file 41598_2017_14419_MOESM1_ESM.doc]

Water-stress induced downsizing of light-harvesting antenna complex protects developing rice seedlings from photo-oxidative damage


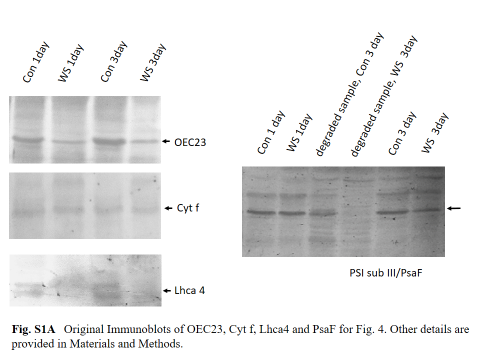
**Vijay K. Dalal and Baishnab C. Tripathy**

Water-stress induced downsizing of light-harvesting antenna complex protects developing rice seedlings from photo-oxidative damage


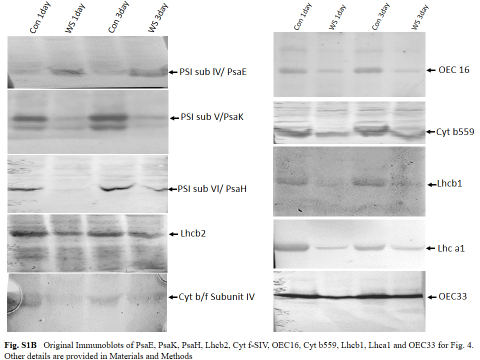
 **Vijay K. Dalal and Baishnab C. Tripathy**
