# Supplementary material for: Water-stress induced downsizing of light-harvesting antenna complex protects developing rice seedlings from photo-oxidative damage
Source: Sci Rep. 2018 Apr 13;8:5955. doi: 10.1038/s41598-017-14419-4 (PMC5899091; doi:10.1038/s41598-017-14419-4)
Supplement: Supplementary file 2 — Supplementary Tables [file 41598_2017_14419_MOESM2_ESM.doc]

| **The gene loci, gene model and other annotations for proteins, retrieved from http://ricedb.plantenergy.uwa.edu.au/**  **Supplementary Table S1: The gene loci, gene model and other annotations for proteins**  **Water-stress induced downsizing of light-harvesting antenna complex protects developing rice seedlings from photo-oxidative damage.**  Vijay K. Dalal and Baishnab C. Tripathy | | | | | | | | |  |  |  |
| --- | --- | --- | --- | --- | --- | --- | --- | --- | --- | --- | --- |
| **IDENTIFIER** | **LOCI** | **MODEL_LOCUS** | **IDS** | **ANNOTATIONS** | **MODEL_SEQUENCES** | **TISSUES** | **EXPRESSED_IN** | **STRESS_EXPRESSION** | **EXP_SHOWN_MOTIFS** | **PREDICTED_LOCATIONS** | **EXP_SHOWN_LOCS** |
| B8B691 | 0 |  | 0 |  |  |  |  |  |  |  |  |
| Q2QW49 | 1 | LOC_Os12g10580.1 | 46 | ribulose bisphosphate carboxylase lar... [v7] | DNA 3916 bp; Upstream 1003 bp; CDS 1476 bp; Peptide 491 aa |  |  |  | 20 upstream DNA binding motif names | cytosol + 1 more from 2 preds | golgi + 2 more in 3 pubs |
| Q2RA00 | 1 | LOC_Os11g07020.2 | 109 | fructose-bisphosphate aldolase, chlor... [v5] | DNA 3107 bp; Upstream 1003 bp; CDS 1155 bp; Peptide 384 aa |  |  |  | 17 upstream DNA binding motif names | chloroplast + 3 more from 4 preds | plastid in 1 pub |
| Q53P96 | 1 | LOC_Os11g07020.3 | 109 | fructose-bisphosphate aldolase, chlor... [v5] | DNA 3107 bp; Upstream 1003 bp; CDS 1092 bp; Peptide 363 aa |  |  |  | 17 upstream DNA binding motif names | chloroplast + 3 more from 4 preds | plastid in 1 pub |
| A3C0Z2 | 0 |  | 0 |  |  |  |  |  |  |  |  |
| A2Y650 | 2 | LOC_Os05g41640.1 | 58 | phosphoglycerate kinase protein, puta... [v7] | DNA 3402 bp; Upstream 1003 bp; CDS 1455 bp; Peptide 484 aa | 41 of 41 | Seed, Germination, Seedling, Germination (N), Seedling (N), Seedling (A/N switches), Leaf, Root, Flower | Control (D,S,C), Drought, Salt, Cold, Control (H), Heat | 40 upstream DNA binding motif names | chloroplast + 2 more from 7 preds | plastid in 1 pub |
| A2Y650 | 2 | LOC_Os05g41640.2 | 66 | phosphoglycerate kinase protein, puta... [v7] | DNA 3402 bp; Upstream 1003 bp; CDS 1455 bp; Peptide 484 aa |  |  |  | 40 upstream DNA binding motif names | chloroplast + 2 more from 7 preds | etioplasts + 1 more in 2 pubs |
| Q0JA18 | 2 | LOC_Os04g52920.1 | 38 | remorin family protein, putative, exp... [v7] | DNA 3248 bp; Upstream 1003 bp; CDS 1599 bp; Peptide 532 aa | 35 of 41 | Seed, Germination, Seedling, Germination (N), Seedling (N), Seedling (A/N switches), Leaf, Root, Flower | Control (D,S,C), Drought, Salt, Cold | 22 upstream DNA binding motif names | chloroplast + 2 more from 5 preds | |
| Q0JA18 | 2 | LOC_Os04g52920.2 | 41 | remorin family protein, putative, exp... [v7] | DNA 3248 bp; Upstream 1003 bp; CDS 1596 bp; Peptide 531 aa |  |  |  | 22 upstream DNA binding motif names | chloroplast + 3 more from 6 preds | |
| Q2R1D9 | 1 | LOC_Os11g39500.1 | 9 | hypothetical protein [v7] | DNA 390 bp; Upstream 1003 bp; CDS 390 bp; Peptide 129 aa | 0 of 41 | Not expressed |  | 30 upstream DNA binding motif names | chloroplast + 1 more from 5 preds | |
| Q2R1D9 | 1 | LOC_Os11g39500.1 | 9 | hypothetical protein [v7] | DNA 390 bp; Upstream 1003 bp; CDS 390 bp; Peptide 129 aa | 0 of 41 | Not expressed |  | 30 upstream DNA binding motif names | chloroplast + 1 more from 5 preds | |
| B8BFR6 | 0 |  | 0 |  |  |  |  |  |  |  |  |
| Q0JKS9 | 1 | LOC_Os01g46340.1 | 49 | chloroplast unusual positioning prote... [v7] | DNA 5836 bp; Upstream 1003 bp; CDS 1833 bp; Peptide 610 aa | 7 of 41 | Seed, Seedling, Leaf, Flower |  | 48 upstream DNA binding motif names | chloroplast + 2 more from 8 preds | |
| Q6L4K2 | 0 |  | 0 |  |  |  |  |  |  |  |  |
| Q5ZE18 | 0 |  | 0 |  |  |  |  |  |  |  |  |
| Q5JKU7 | 0 |  | 0 |  |  |  |  |  |  |  |  |
| Q9FW56 | 0 |  | 0 |  |  |  |  |  |  |  |  |
| Q67UQ5 | 0 |  | 0 |  |  |  |  |  |  |  |  |
| B8B691 | 0 |  | 0 |  |  |  |  |  |  |  |  |
| Q2QLY7 | 1 | LOC_Os12g42860.1 | 41 | 2-aminoethanethiol dioxygenase, putat... [v7] | DNA 2711 bp; Upstream 1003 bp; CDS 897 bp; Peptide 298 aa | 41 of 41 | Seed, Germination, Seedling, Germination (N), Seedling (N), Seedling (A/N switches), Leaf, Root, Flower | Control (D,S,C), Drought, Salt, Cold, Control (H), Heat | 19 upstream DNA binding motif names | nucleus from 3 preds | |
| A2WQA2 | 0 |  | 0 |  |  |  |  |  |  |  |  |
| Q6EPW4 | 1 | LOC_Os02g33220.1 | 8 | diap1 protein, putative, expressed [v7] | DNA 450 bp; Upstream 1003 bp; CDS 450 bp; Peptide 149 aa | 0 of 41 | Not expressed |  | 18 upstream DNA binding motif names | chloroplast + 3 more from 8 preds | |
| Q6EPW4 | 1 | LOC_Os02g33220.1 | 8 | diap1 protein, putative, expressed [v7] | DNA 450 bp; Upstream 1003 bp; CDS 450 bp; Peptide 149 aa | 0 of 41 | Not expressed |  | 18 upstream DNA binding motif names | chloroplast + 3 more from 8 preds | |
| Q655X0 | 1 | LOC_Os06g45510.1 | 39 | thioredoxin, putative, expressed [v7] | DNA 2787 bp; Upstream 1003 bp; CDS 519 bp; Peptide 172 aa | 41 of 41 | Seed, Germination, Seedling, Germination (N), Seedling (N), Seedling (A/N switches), Leaf, Root, Flower | Control (D,S,C), Drought, Salt, Cold, Control (H), Heat | 34 upstream DNA binding motif names | mitochondrion + 4 more from 9 preds | |
| Q5JNB0 | 1 | LOC_Os01g74650.3 | 190 | cysteine synthase, mitochondrial prec... [v7] | DNA 6572 bp; Upstream 1003 bp; CDS 1185 bp; Peptide 394 aa |  |  |  | 44 upstream DNA binding motif names | chloroplast + 3 more from 8 preds | etioplasts + 1 more in 2 pubs |
| Q10J19 | 1 | LOC_Os03g32290.1 | 8 | expressed protein [v7] | DNA 2047 bp; Upstream 1003 bp; CDS 321 bp; Peptide 106 aa | 0 of 41 | Not expressed |  | 28 upstream DNA binding motif names | mitochondrion + 3 more from 6 preds | |
| Q10J19 | 1 | LOC_Os03g32290.1 | 8 | expressed protein [v7] | DNA 2047 bp; Upstream 1003 bp; CDS 321 bp; Peptide 106 aa | 0 of 41 | Not expressed |  | 28 upstream DNA binding motif names | mitochondrion + 3 more from 6 preds | |
| Q69ML5 | 1 | LOC_Os09g06160.1 | 11 | retrotransposon protein, putative, Ty... [v7] | DNA 868 bp; Upstream 1003 bp; CDS 534 bp; Peptide 177 aa |  |  |  | 46 upstream DNA binding motif names | mitochondrion + 1 more from 5 preds | |
| Q69ML5 | 1 | LOC_Os09g06160.1 | 11 | retrotransposon protein, putative, Ty... [v7] | DNA 868 bp; Upstream 1003 bp; CDS 534 bp; Peptide 177 aa |  |  |  | 46 upstream DNA binding motif names | mitochondrion + 1 more from 5 preds | |
| B9EUG1 | 0 |  | 0 |  |  |  |  |  |  |  |  |
| Q0JF20 | 1 | LOC_Os04g09430.1 | 38 | cytochrome P450, putative, expressed [v7] | DNA 2654 bp; Upstream 1003 bp; CDS 762 bp; Peptide 253 aa | 0 of 41 | Not expressed |  | 29 upstream DNA binding motif names | mitochondrion + 1 more from 3 preds | |
| Q7Y1I1 | 1 | LOC_Os03g58270.1 | 19 | retrotransposon protein, putative, un... [v7] | DNA 4068 bp; Upstream 1003 bp; CDS 3573 bp; Peptide 1190 aa |  |  |  | 36 upstream DNA binding motif names | cytosol + 2 more from 4 preds | |
| Q01KS5 | 0 |  | 0 |  |  |  |  |  |  |  |  |
| Q5VP50 | 1 | LOC_Os06g05359.1 | 42 | NBS-LRR disease resistance protein, p... [v7] | DNA 8393 bp; Upstream 1003 bp; CDS 3081 bp; Peptide 1026 aa | 16 of 41 | Seed, Germination, Seedling, Leaf, Root, Flower | Control (D,S,C), Salt, Cold, Control (H), Heat | 34 upstream DNA binding motif names | cytosol + 3 more from 4 preds | |
| Q84Z86 | 2 | LOC_Os08g40630.1 | 61 | mTERF domain containing protein, expr... [v7] | DNA 2723 bp; Upstream 1003 bp; CDS 1911 bp; Peptide 636 aa | 37 of 41 | Seed, Germination, Seedling, Germination (N), Seedling (N), Seedling (A/N switches), Leaf, Root, Flower | Control (D,S,C), Cold, Control (H), Heat | 46 upstream DNA binding motif names | mitochondrion + 2 more from 7 preds | mitochondrion + 1 more in 1 pub |
| Q84Z86 | 2 | LOC_Os08g40630.2 | 62 | mTERF domain containing protein, expr... [v7] | DNA 2723 bp; Upstream 1003 bp; CDS 1911 bp; Peptide 636 aa |  |  |  | 46 upstream DNA binding motif names | mitochondrion + 2 more from 7 preds | mitochondrion + 1 more in 1 pub |
| A2XB47 | 1 | LOC_Os02g57550.1 | 23 | ZOS2-18 - C2H2 zinc finger protein, e... [v7] | DNA 588 bp; Upstream 1003 bp; CDS 588 bp; Peptide 195 aa | 0 of 41 | Not expressed |  | 34 upstream DNA binding motif names | nucleus from 2 preds | |
| Q5Z6Q9 | 0 |  | 0 |  |  |  |  |  |  |  |  |
| B8AMQ0 | 0 |  | 0 |  |  |  |  |  |  |  |  |
| Q0J0U5 | 1 | LOC_Os09g31000.1 | 40 | EF hand family protein, expressed [v7] | DNA 840 bp; Upstream 1003 bp; CDS 417 bp; Peptide 138 aa | 29 of 41 | Seed, Germination, Seedling, Germination (N), Seedling (N), Seedling (A/N switches), Leaf, Root, Flower | Drought, Salt | 45 upstream DNA binding motif names | chloroplast + 1 more from 2 preds | cytoplasm + 1 more in 1 pub |
| Q10L79 | 1 | LOC_Os03g24160.1 | 40 | phosphatidylinositol-4-phosphate 5-ki... [v7] | DNA 4485 bp; Upstream 1003 bp; CDS 2268 bp; Peptide 755 aa | 17 of 41 | Seed, Germination, Seedling, Seedling (A/N switches), Leaf, Root, Flower | Cold, Control (H), Heat | 36 upstream DNA binding motif names | cytosol + 1 more from 2 preds | |
| Q10L79 | 1 | LOC_Os03g24160.1 | 40 | phosphatidylinositol-4-phosphate 5-ki... [v7] | DNA 4485 bp; Upstream 1003 bp; CDS 2268 bp; Peptide 755 aa | 17 of 41 | Seed, Germination, Seedling, Seedling (A/N switches), Leaf, Root, Flower | Cold, Control (H), Heat | 36 upstream DNA binding motif names | cytosol + 1 more from 2 preds | |
| Q6Z8K7 | 3 | LOC_Os02g51470.1 | 40 | ATP synthase F1, delta subunit family... [v7] | DNA 1638 bp; Upstream 1003 bp; CDS 732 bp; Peptide 243 aa | 40 of 41 | Seed, Germination, Seedling, Germination (N), Seedling (N), Seedling (A/N switches), Leaf, Root, Flower | Control (D,S,C), Drought, Salt, Cold, Control (H), Heat | 33 upstream DNA binding motif names | mitochondrion + 2 more from 8 preds | plastid in 1 pub |
| Q6Z8K7 | 3 | LOC_Os02g51470.2 | 40 | ATP synthase F1, delta subunit family... [v7] | DNA 1638 bp; Upstream 1003 bp; CDS 732 bp; Peptide 243 aa |  |  |  | 33 upstream DNA binding motif names | mitochondrion + 2 more from 8 preds | etioplasts + 1 more in 2 pubs |
| Q6Z8K7 | 3 | LOC_Os02g51470.3 | 42 | ATP synthase F1, delta subunit family... [v7] | DNA 1638 bp; Upstream 1003 bp; CDS 732 bp; Peptide 243 aa |  |  |  | 33 upstream DNA binding motif names | mitochondrion + 2 more from 8 preds | etioplasts + 1 more in 2 pubs |
| Q6ZGJ8 | 1 | LOC_Os02g52940.2 | 107 | soluble inorganic pyrophosphatase, pu... [v7] | DNA 3677 bp; Upstream 1003 bp; CDS 861 bp; Peptide 286 aa |  |  |  | 42 upstream DNA binding motif names | chloroplast + 3 more from 8 preds | plastid in 1 pub |
| Q943W1 | 1 | LOC_Os01g31690.1 | 535 | oxygen-evolving enhancer protein 1, c... [v7] | DNA 1506 bp; Upstream 1003 bp; CDS 1002 bp; Peptide 333 aa | 38 of 41 | Seed, Germination, Seedling, Germination (N), Seedling (N), Seedling (A/N switches), Leaf, Root, Flower | Control (D,S,C), Drought, Salt, Cold, Control (H), Heat | 52 upstream DNA binding motif names | chloroplast + 2 more from 7 preds | etioplasts in 1 pub |
| Q0J032 | 1 | LOC_Os09g36300.1 | 143 | OsLonP4 - Putative Lon protease homol... [v7] | DNA 7022 bp; Upstream 1003 bp; CDS 2655 bp; Peptide 884 aa | 41 of 41 | Seed, Germination, Seedling, Germination (N), Seedling (N), Seedling (A/N switches), Leaf, Root, Flower | Control (D,S,C), Drought, Salt, Cold, Control (H), Heat | 48 upstream DNA binding motif names | chloroplast + 3 more from 4 preds | |
| Q10N21 | 2 | LOC_Os03g17690.2 | 192 | OsAPx1 - Cytosolic Ascorbate Peroxida... [v7] | DNA 3421 bp; Upstream 1003 bp; CDS 576 bp; Peptide 191 aa |  |  |  | 52 upstream DNA binding motif names | cytosol from 2 preds | etioplasts in 1 pub |
| Q10N21 | 1 | LOC_Os03g17690.1 | 199 | OsAPx1 - Cytosolic Ascorbate Peroxida... [v7] | DNA 3421 bp; Upstream 1003 bp; CDS 753 bp; Peptide 250 aa | 41 of 41 | Seed, Germination, Seedling, Germination (N), Seedling (N), Seedling (A/N switches), Leaf, Root, Flower | Control (D,S,C), Drought, Salt, Cold, Control (H), Heat | 48 upstream DNA binding motif names | cytosol from 2 preds | etioplasts in 1 pub |
| Q65XA0 | 1 | LOC_Os05g02530.1 | 267 | glutathione S-transferase, N-terminal... [v7] | DNA 3679 bp; Upstream 1003 bp; CDS 894 bp; Peptide 297 aa | 41 of 41 | Seed, Germination, Seedling, Germination (N), Seedling (N), Seedling (A/N switches), Leaf, Root, Flower | Control (D,S,C), Drought, Salt, Cold, Control (H), Heat | 22 upstream DNA binding motif names | chloroplast + 2 more from 6 preds | mitochondrion in 1 pub |
| Q7X7H4 | 1 | LOC_Os04g45580.1 | 61 | kinesin motor domain containing prote... [v7] | DNA 6449 bp; Upstream 1003 bp; CDS 2838 bp; Peptide 945 aa | 41 of 41 | Seed, Germination, Seedling, Germination (N), Seedling (N), Seedling (A/N switches), Leaf, Root, Flower | Control (D,S,C), Drought, Salt, Cold, Control (H), Heat | 47 upstream DNA binding motif names | nucleus + 2 more from 4 preds | |
| A2YTG8 | 1 | LOC_Os08g18880.1 | 55 | WD domain, G-beta repeat domain conta... [v7] | DNA 3602 bp; Upstream 1003 bp; CDS 1350 bp; Peptide 449 aa | 2 of 41 | Seed, Leaf |  | 18 upstream DNA binding motif names | cytoskeleton + 1 more from 2 preds | |
| Q6F361 | 1 | LOC_Os05g49880.1 | 86 | lactate/malate dehydrogenase, putativ... [v7] | DNA 3991 bp; Upstream 1003 bp; CDS 1023 bp; Peptide 340 aa | 41 of 41 | Seed, Germination, Seedling, Germination (N), Seedling (N), Seedling (A/N switches), Leaf, Root, Flower | Control (D,S,C), Drought, Salt, Cold, Control (H), Heat | 41 upstream DNA binding motif names | mitochondrion from 6 preds | plastid + 1 more in 2 pubs |
| Q6ZHF3 | 1 | LOC_Os02g53270.1 | 49 | expressed protein [v7] | DNA 8934 bp; Upstream 1003 bp; CDS 3495 bp; Peptide 1164 aa | 41 of 41 | Seed, Germination, Seedling, Germination (N), Seedling (N), Seedling (A/N switches), Leaf, Root, Flower | Control (D,S,C), Drought, Salt, Cold, Control (H), Heat | 23 upstream DNA binding motif names | chloroplast + 2 more from 5 preds | |
| Q75M32 | 1 | LOC_Os05g01270.1 | 133 | peptidyl-prolyl cis-trans isomerase, ... [v7] | DNA 2866 bp; Upstream 1003 bp; CDS 753 bp; Peptide 250 aa | 41 of 41 | Seed, Germination, Seedling, Germination (N), Seedling (N), Seedling (A/N switches), Leaf, Root, Flower | Control (D,S,C), Drought, Salt, Cold, Control (H), Heat | 16 upstream DNA binding motif names | chloroplast + 3 more from 8 preds | etioplasts in 1 pub |
| Q94JA2 | 1 | LOC_Os01g46070.1 | 129 | lactate/malate dehydrogenase, putativ... [v7] | DNA 3766 bp; Upstream 1003 bp; CDS 1023 bp; Peptide 340 aa | 41 of 41 | Seed, Germination, Seedling, Germination (N), Seedling (N), Seedling (A/N switches), Leaf, Root, Flower | Control (D,S,C), Drought, Salt, Cold, Control (H), Heat | 14 upstream DNA binding motif names | mitochondrion + 1 more from 6 preds | mitochondrion in 5 pubs |
| Q6YUR8 | 1 | LOC_Os02g02870.1 | 106 | glycine-rich protein 2, putative, exp... [v7] | DNA 1342 bp; Upstream 1003 bp; CDS 726 bp; Peptide 241 aa | 41 of 41 | Seed, Germination, Seedling, Germination (N), Seedling (N), Seedling (A/N switches), Leaf, Root, Flower | Control (D,S,C), Drought, Salt, Cold, Control (H), Heat | 76 upstream DNA binding motif names | mitochondrion + 2 more from 6 preds | etioplasts in 1 pub |
| Q9SXP2 | 1 | LOC_Os06g29180.1 | 167 | erythronate-4-phosphate dehydrogenase... [v7] | DNA 3753 bp; Upstream 1003 bp; CDS 1131 bp; Peptide 376 aa | 37 of 41 | Seed, Germination, Seedling, Germination (N), Seedling (N), Seedling (A/N switches), Leaf, Root, Flower | Control (D,S,C), Drought, Salt, Cold, Control (H), Heat | 38 upstream DNA binding motif names | mitochondrion + 1 more from 7 preds | mitochondrion in 3 pubs |
| Q7XPK1 | 2 | LOC_Os04g57010.2 | 79 | zinc finger C-x8-C-x5-C-x3-H type fam... [v7] | DNA 3299 bp; Upstream 1003 bp; CDS 663 bp; Peptide 220 aa |  |  |  | 20 upstream DNA binding motif names | cytosol + 1 more from 2 preds | |
| Q7XPK1 | 1 | LOC_Os04g57010.1 | 83 | zinc finger C-x8-C-x5-C-x3-H type fam... [v7] | DNA 3299 bp; Upstream 1003 bp; CDS 930 bp; Peptide 309 aa | 41 of 41 | Seed, Germination, Seedling, Germination (N), Seedling (N), Seedling (A/N switches), Leaf, Root, Flower | Control (D,S,C), Drought, Salt, Cold, Control (H), Heat | 30 upstream DNA binding motif names | cytosol + 2 more from 3 preds | |
| Q851L5 | 1 | LOC_Os03g62060.1 | 53 | hydrolase, putative, expressed [v7] | DNA 2833 bp; Upstream 1003 bp; CDS 1254 bp; Peptide 417 aa | 13 of 41 | Seed, Germination, Seedling, Seedling (A/N switches), Leaf, Root | Control (D,S,C), Drought, Salt, Cold | 27 upstream DNA binding motif names | cytosol + 1 more from 4 preds | etioplasts in 1 pub |
| Q851L5 | 1 | LOC_Os03g62060.1 | 53 | hydrolase, putative, expressed [v7] | DNA 2833 bp; Upstream 1003 bp; CDS 1254 bp; Peptide 417 aa | 13 of 41 | Seed, Germination, Seedling, Seedling (A/N switches), Leaf, Root | Control (D,S,C), Drought, Salt, Cold | 27 upstream DNA binding motif names | cytosol + 1 more from 4 preds | etioplasts in 1 pub |
| Q7XDC8 | 1 | LOC_Os10g33800.1 | 161 | lactate/malate dehydrogenase, putativ... [v7] | DNA 4033 bp; Upstream 1003 bp; CDS 999 bp; Peptide 332 aa | 41 of 41 | Seed, Germination, Seedling, Germination (N), Seedling (N), Seedling (A/N switches), Leaf, Root, Flower | Control (D,S,C), Drought, Salt, Cold, Control (H), Heat | 69 upstream DNA binding motif names | cytosol + 1 more from 2 preds | |
| Q7XDC8 | 3 | LOC_Os10g33800.2 | 157 | lactate/malate dehydrogenase, putativ... [v7] | DNA 4033 bp; Upstream 1003 bp; CDS 951 bp; Peptide 316 aa |  |  |  | 69 upstream DNA binding motif names | cytosol + 1 more from 2 preds | |
| Q7XDC8 | 3 | LOC_Os10g33800.3 | 157 | lactate/malate dehydrogenase, putativ... [v7] | DNA 4033 bp; Upstream 1003 bp; CDS 660 bp; Peptide 219 aa |  |  |  | 69 upstream DNA binding motif names | chloroplast + 1 more from 2 preds | |
| Q2QRD6 | 1 | LOC_Os12g27930.1 | 19 | expressed protein [v7] | DNA 896 bp; Upstream 1003 bp; CDS 384 bp; Peptide 127 aa | 2 of 41 | Seed |  | 35 upstream DNA binding motif names | secretory SP + 2 more from 6 preds | |
| Q2QRD6 | 1 | LOC_Os12g27930.1 | 19 | expressed protein [v7] | DNA 896 bp; Upstream 1003 bp; CDS 384 bp; Peptide 127 aa | 2 of 41 | Seed |  | 35 upstream DNA binding motif names | secretory SP + 2 more from 6 preds | |
| Q5QNA8 | 1 | LOC_Os01g11000.1 | 15 | transposon protein, putative, unclass... [v7] | DNA 4094 bp; Upstream 1003 bp; CDS 879 bp; Peptide 292 aa | 0 of 41 | Not expressed |  | 49 upstream DNA binding motif names | chloroplast + 2 more from 3 preds | |
| Q5QNA8 | 1 | LOC_Os01g11000.1 | 15 | transposon protein, putative, unclass... [v7] | DNA 4094 bp; Upstream 1003 bp; CDS 879 bp; Peptide 292 aa | 0 of 41 | Not expressed |  | 49 upstream DNA binding motif names | chloroplast + 2 more from 3 preds | |
| Q69YD6 | 0 |  | 0 |  |  |  |  |  |  |  |  |
| Q6EQ30 | 0 |  | 0 |  |  |  |  |  |  |  |  |
| A2WPN7 | 0 |  | 0 |  |  |  |  |  |  |  |  |
| T02B73 | 0 |  | 0 |  |  |  |  |  |  |  |  |

| **The gene loci and other annotations for identified proteins, retrieved from Uniprot** | | | | | | | | | | |
| --- | --- | --- | --- | --- | --- | --- | --- | --- | --- | --- |
| **GI** | **Entry** | **Entry name** | **Status** | **Protein names** | ***Organism*** | **Length** |  | **Locus/Gene name** | |  |
| 108862318 | Q2QW49 | Q2QW49_ORYSJ | unreviewed | Ribulose bisphosphate carboxylase large chain, putative | *Oryza sativa subsp. japonica (Rice)* | 491 |  | LOC_Os12g10580 |  |  |
| 108864048 | Q2RA00 | Q2RA00_ORYSJ | unreviewed | Fructose-bisphosphate aldolase (EC 4.1.2.13) | *Oryza sativa subsp. japonica (Rice)* | 384 |  | LOC_Os11g07020 |  |  |
| 62732954 | Q53P96 | Q53P96_ORYSJ | unreviewed | Fructose-bisphosphate aldolase (EC 4.1.2.13) | *Oryza sativa subsp. japonica (Rice)* | 363 |  | LOC_Os11g07020 |  |  |
| 125606445 | A3C0Z2 | A3C0Z2_ORYSJ | unreviewed | Triosephosphate isomerase (EC 5.3.1.1) | *Oryza sativa subsp. japonica (Rice)* | 245 |  | OsJ_30136 |  |  |
| 125552851 | A2Y650 | A2Y650_ORYSI | unreviewed | Phosphoglycerate kinase (EC 2.7.2.3) | *Oryza sativa subsp. indica (Rice)* | 284 |  | OsI_20474 |  |  |
| 115460610 | Q0JA18 | Q0JA18_ORYSJ | unreviewed | Os04g0620200 protein (Fragment) | *Oryza sativa subsp. japonica (Rice)* | 284 |  | Os04g0620200 |  |  |
| 77551932 | Q2R1D9 | Q2R1D9_ORYSJ | unreviewed | Uncharacterized protein | *Oryza sativa subsp. japonica (Rice)* | 129 | No domain | LOC_Os11g39500 | OsJ_02020 |  |
| 218184148 | B8BFR6 | B8BFR6_ORYSI | unreviewed | Uncharacterized protein | *Oryza sativa subsp. indica (Rice)* | 104 |  | OsI_32755 |  |  |
| 115438911 | Q0JKS9 | Q0JKS9_ORYSJ | unreviewed | Os01g0652000 protein | *Oryza sativa subsp. japonica (Rice)* | 97 | No domain | Os01g0652000 |  |  |
| 47900462 | Q6L4K2 | Q6L4K2_ORYSJ | unreviewed | Putative uncharacterized protein OSJNBb0079L11.15 | *Oryza sativa subsp. japonica (Rice)* | 112 | Transmembrane domain | OSJNBb0079L11.15 | |  |
| 53791471 | Q5ZE18 | Q5ZE18_ORYSJ | unreviewed | Putative uncharacterized protein P0410E03.12 | *Oryza sativa subsp. japonica (Rice)* | 60 | small size, No domain | P0410E03.12 |  |  |
| 57899958 | Q5JKU7 | Q5JKU7_ORYSJ | unreviewed | Putative uncharacterized protein P0439E07.9 | *Oryza sativa subsp. japonica (Rice)* | 69 | small size, No domain | P0439E07.9 |  |  |
| 10440615 | Q9FW56 | Q9FW56_ORYSJ | unreviewed | Putative uncharacterized protein OSJNBb0094K03.9 | *Oryza sativa subsp. japonica (Rice)* | 138 | No domain | OSJNBb0094K03.9 | |  |
| 51534990 | Q67UQ5 | Q67UQ5_ORYSJ | unreviewed | Putative uncharacterized protein P0453H04.33 | *Oryza sativa subsp. japonica (Rice)* | 146 |  | P0453H04.33 |  |  |
| 218199617 | B8B691 | B8B691_ORYSI | unreviewed | Putative uncharacterized protein | *Oryza sativa subsp. indica (Rice)* | 237 |  | OsI_26020 |  |  |
| 115489646 | Q2QLY7 | Q2QLY7_ORYSJ | unreviewed | (RAP Annotation release2) Protein of unknown function DUF1637 family protein (Expressed protein) (Os12g0623600 protein) | *Oryza sativa subsp. japonica (Rice)* | 298 |  | LOC_Os12g42860 | Os12g0623600 OsJ_36924 | |
| 125526034 | A2WQA2 | A2WQA2_ORYSI | unreviewed | Putative uncharacterized protein | *Oryza sativa subsp. indica (Rice)* | 116 | No domain | OsI_02030 |  |  |
| 50251877 | Q6EPW4 | Q6EPW4_ORYSJ | unreviewed | Putative uncharacterized protein B1136H02.30 (Putative uncharacterized protein OJ1112_G07.17) | *Oryza sativa subsp. japonica (Rice)* | 149 |  | OJ1112_G07.17 | B1136H02.30 |  |
| 222636049 | Q655X0 | TRXO_ORYSJ | reviewed | Thioredoxin O, mitochondrial (OsTrxo1) (OsTrx22) | *Oryza sativa subsp. japonica (Rice)* | 174 |  | LOC_Os06g45510 | Os06g0665900 | OsJ_22283 P0473H04.30 P0637D03.1 |
| 115442595 | Q5JNB0 | Q5JNB0_ORYSJ | unreviewed | Cysteine synthase (EC 2.5.1.47) | *Oryza sativa subsp. japonica (Rice)* | 394 |  | Os01g0978100 | OsJ_04976 | P0020E09.29 |
| 108709024 | Q10J19 | Q10J19_ORYSJ | unreviewed | Putative uncharacterized protein | *Oryza sativa subsp. japonica (Rice)* | 106 |  | LOC_Os03g32290 |  |  |
| 50725174 | Q69ML5 | Q69ML5_ORYSJ | unreviewed | Aminotransferase-like | *Oryza sativa subsp. japonica (Rice)* | 177 |  | OSJNBa0017O03.4 | |  |
| 222619550 | B9EUG1 | B9EUG1_ORYSJ | unreviewed | Uncharacterized protein | *Oryza sativa subsp. japonica (Rice)* | 732 |  | OsJ_04099 |  |  |
| 115457106 | Q0JF20 | Q0JF20_ORYSJ | unreviewed | Os04g0174100 protein | *Oryza sativa subsp. japonica (Rice)* | 253 |  | Os04g0174100 |  |  |
| 31126757 | Q7Y1I1 | Q7Y1I1_ORYSJ | unreviewed | Putative gag-pol polyprotein | *Oryza sativa subsp. japonica (Rice)* | 1182 |  | OSJNBa0094F01.17 | |  |
| 116309588 | Q01KS5 | Q01KS5_ORYSA | unreviewed | OSIGBa0146I21.3 protein | *Oryza sativa (Rice)* | 684 |  | OSIGBa0146I21.3 |  |  |
| 115466384 | Q5VP50 | Q5VP50_ORYSJ | unreviewed | Os06g0146100 protein (Putative NBS-LRR disease resistance protein) | *Oryza sativa subsp. japonica (Rice)* | 1026 |  | Os06g0146100 | P0036F10.15 OSJNBa0007O20.32 | OsJ_20116 |
| 115477308 | Q84Z86 | Q84Z86_ORYSJ | unreviewed | Os08g0518200 protein (Putative uncharacterized protein P0700D12.120) (cDNA clone:001-124-E07, full insert sequence) (cDNA clone:J033081L13, full insert sequence) | *Oryza sativa subsp. japonica (Rice)* | 636 |  | Os08g0518200 | P0700D12.120 | OsJ_27943 |
| 125541662 | A2XB47 | A2XB47_ORYSI | unreviewed | Putative uncharacterized protein | *Oryza sativa subsp. indica (Rice)* | 195 |  | OsI_09486 |  |  |
| 3793156 | Q5Z6Q9 | Q5Z6Q9_ORYSJ | unreviewed | Putative uncharacterized protein OSJNBa0037N01.45 | *Oryza sativa subsp. japonica (Rice)* | 107 |  | OSJNBa0037N01.45 | |  |
| 218194019 | B8AMQ0 | B8AMQ0_ORYSI | unreviewed | Putative uncharacterized protein | *Oryza sativa subsp. indica (Rice)* | 443 |  | OsI_14142 |  |  |
| 115479825 | Q0J0U5 | Q0J0U5_ORYSJ | unreviewed | Os09g0483100 protein | *Oryza sativa subsp. japonica (Rice)* | 138 |  | Os09g0483100 | OsJ_29803 |  |
| 108708236 | Q10L79 | Q10L79_ORYSJ | unreviewed | Os03g0356582 protein (Phosphatidylinositol-4-phosphate 5-kinase 4, putative, expressed) (cDNA clone:J023019H02, full insert sequence) | *Oryza sativa subsp. japonica (Rice)* | 755 |  | LOC_Os03g24160 | Os03g0356582 |  |

**Supplementary Table S2: a) Gene Ontology enrichment; b) proteins (IDs) annotated/ recognized, and c) un-annotated/not-recognized by Ricearray for GO terms/names**

| a) Gene Ontology enrichment with 27 annotated/recognized proteins IDs was performed with Ricearry (http://www.ricearray.org/analysis/go_enrichment.php) | | | | | | | | |  |
| --- | --- | --- | --- | --- | --- | --- | --- | --- | --- |
| **GO enrichment analysis results.** | | | | | | | | |  |
| **GO ID** | **GO Name** | **GO Level** | **Ref Total** | **Ref Number** | **Query Total** | **Query Number** | **Query Exp** | **Hyper p value** |  |
| GO:0006096 | glycolysis | 5 | 39571 | 124 | 63 | 5 | 0.1974 | 0.0000 |  |
| GO:0006099 | tricarboxylic acid cycle | 7, 8 | 39571 | 33 | 63 | 3 | 0.0525 | 0.0000 |  |
| GO:0006108 | malate metabolic process | 8 | 39571 | 23 | 63 | 3 | 0.0366 | 0.0000 |  |
| GO:0044262 | cellular carbohydrate metabolic process | 4 | 39571 | 18 | 63 | 3 | 0.0287 | 0.0000 |  |
| GO:0055114 | oxidation reduction | 3 | 39571 | 1199 | 63 | 7 | 1.9089 | 0.0023 |  |
| GO:0019344 | cysteine biosynthetic process | 7 | 39571 | 5 | 63 | 1 | 0.0080 | 0.0079 |  |
| GO:0042549 | photosystem II stabilization | 7, 8 | 39571 | 6 | 63 | 1 | 0.0096 | 0.0095 |  |
| GO:0015977 | carbon fixation | 4 | 39571 | 13 | 63 | 1 | 0.0207 | 0.0203 |  |
| GO:0042744 | hydrogen peroxide catabolic process | 6 | 39571 | 13 | 63 | 1 | 0.0207 | 0.0203 |  |
| GO:0006535 | cysteine biosynthetic process from serine | 8 | 39571 | 17 | 63 | 1 | 0.0271 | 0.0264 |  |
| GO:0046488 | phosphatidylinositol metabolic process | 6 | 39571 | 18 | 63 | 1 | 0.0287 | 0.0279 |  |
| GO:0015074 | DNA integration | 6 | 39571 | 3113 | 63 | 1 | 4.9561 | 0.0307 |  |
| GO:0006796 | phosphate metabolic process | 5 | 39571 | 31 | 63 | 1 | 0.0494 | 0.0471 |  |

| **b) GO terms mapped onto the query.** | | | | | |
| --- | --- | --- | --- | --- | --- |
| **Locus ID** | **RGAP Ver 6 Annotation** | **GO ID** | **GO Name** | **GO Type** | **Evidence Type** |
| LOC_Os01g31690 | oxygen-evolving enhancer protein 1, chloroplast precursor, putative, expressed | GO:0015979 | photosynthesis | biological_process | IEA |
| LOC_Os01g31690 | oxygen-evolving enhancer protein 1, chloroplast precursor, putative, expressed | GO:0042549 | photosystem II stabilization | biological_process | IEA |
| LOC_Os01g46070 | lactate/malate dehydrogenase, putative, expressed | GO:0055114 | oxidation reduction | biological_process | IEA |
| LOC_Os01g46070 | lactate/malate dehydrogenase, putative, expressed | GO:0044262 | cellular carbohydrate metabolic process | biological_process | IEA |
| LOC_Os01g46070 | lactate/malate dehydrogenase, putative, expressed | GO:0008152 | metabolic process | biological_process | IEA |
| LOC_Os01g46070 | lactate/malate dehydrogenase, putative, expressed | GO:0006108 | malate metabolic process | biological_process | IEA |
| LOC_Os01g46070 | lactate/malate dehydrogenase, putative, expressed | GO:0006099 | tricarboxylic acid cycle | biological_process | IEA |
| LOC_Os01g46070 | lactate/malate dehydrogenase, putative, expressed | GO:0006096 | glycolysis | biological_process | IEA |
| LOC_Os01g46070 | lactate/malate dehydrogenase, putative, expressed | GO:0005975 | carbohydrate metabolic process | biological_process | IEA |
| LOC_Os01g74650 | cysteine synthase, mitochondrial precursor, putative, expressed | GO:0006535 | cysteine biosynthetic process from serine | biological_process | IEA |
| LOC_Os01g74650 | cysteine synthase, mitochondrial precursor, putative, expressed | GO:0008152 | metabolic process | biological_process | IEA |
| LOC_Os01g74650 | cysteine synthase, mitochondrial precursor, putative, expressed | GO:0008652 | cellular amino acid biosynthetic process | biological_process | IEA |
| LOC_Os01g74650 | cysteine synthase, mitochondrial precursor, putative, expressed | GO:0019344 | cysteine biosynthetic process | biological_process | IEA |
| LOC_Os02g02870 | glycine-rich protein 2, putative, expressed | GO:0006355 | regulation of transcription, DNA-dependent | biological_process | IEA |
| LOC_Os02g02870 | glycine-rich protein 2, putative, expressed | GO:0051260 | protein homooligomerization | biological_process | IEA |
| LOC_Os02g33220 | diap1 protein, putative | GO:0006334 | nucleosome assembly | biological_process | IEA |
| LOC_Os02g51470 | ATP synthase F1, delta subunit family protein, putative, expressed | GO:0006754 | ATP biosynthetic process | biological_process | IEA |
| LOC_Os02g51470 | ATP synthase F1, delta subunit family protein, putative, expressed | GO:0006810 | transport | biological_process | IEA |
| LOC_Os02g51470 | ATP synthase F1, delta subunit family protein, putative, expressed | GO:0006811 | ion transport | biological_process | IEA |
| LOC_Os02g51470 | ATP synthase F1, delta subunit family protein, putative, expressed | GO:0015986 | ATP synthesis coupled proton transport | biological_process | IEA |
| LOC_Os02g52940 | soluble inorganic pyrophosphatase, putative, expressed | GO:0006796 | phosphate metabolic process | biological_process | IEA |
| LOC_Os02g53270 | expressed protein | GO:0006396 | RNA processing | biological_process | IEA |
| LOC_Os02g57550 | ZOS2-18 - C2H2 zinc finger protein | GO:0050826 | response to freezing | biological_process | IEA |
| LOC_Os02g57550 | ZOS2-18 - C2H2 zinc finger protein | GO:0042309 | homoiothermy | biological_process | IEA |
| LOC_Os03g17690 | OsAPx1 - Cytosolic Ascorbate Peroxidase encoding gene 1-8, expressed | GO:0006950 | response to stress | biological_process | IEA |
| LOC_Os03g17690 | OsAPx1 - Cytosolic Ascorbate Peroxidase encoding gene 1-8, expressed | GO:0006979 | response to oxidative stress | biological_process | IEA |
| LOC_Os03g17690 | OsAPx1 - Cytosolic Ascorbate Peroxidase encoding gene 1-8, expressed | GO:0042744 | hydrogen peroxide catabolic process | biological_process | IEA |
| LOC_Os03g17690 | OsAPx1 - Cytosolic Ascorbate Peroxidase encoding gene 1-8, expressed | GO:0055114 | oxidation reduction | biological_process | IEA |
| LOC_Os03g24160 | phosphatidylinositol-4-phosphate 5-kinase, putative, expressed | GO:0007186 | G-protein coupled receptor protein signaling pathway | biological_process | IEA |
| LOC_Os03g24160 | phosphatidylinositol-4-phosphate 5-kinase, putative, expressed | GO:0046488 | phosphatidylinositol metabolic process | biological_process | IEA |
| LOC_Os03g58270 | retrotransposon protein, putative, unclassified | GO:0015074 | DNA integration | biological_process | IEA |
| LOC_Os03g62060 | hydrolase, putative, expressed | GO:0006508 | proteolysis | biological_process | IEA |
| LOC_Os04g09430 | cytochrome P450, putative, expressed | GO:0055114 | oxidation reduction | biological_process | IEA |
| LOC_Os04g45580 | kinesin motor domain containing protein, expressed | GO:0007018 | microtubule-based movement | biological_process | IEA |
| LOC_Os05g01270 | peptidyl-prolyl cis-trans isomerase, putative, expressed | GO:0006457 | protein folding | biological_process | IEA |
| LOC_Os05g41640 | phosphoglycerate kinase protein, putative, expressed | GO:0006096 | glycolysis | biological_process | IEA |
| LOC_Os05g49880 | lactate/malate dehydrogenase, putative, expressed | GO:0055114 | oxidation reduction | biological_process | IEA |
| LOC_Os05g49880 | lactate/malate dehydrogenase, putative, expressed | GO:0044262 | cellular carbohydrate metabolic process | biological_process | IEA |
| LOC_Os05g49880 | lactate/malate dehydrogenase, putative, expressed | GO:0008152 | metabolic process | biological_process | IEA |
| LOC_Os05g49880 | lactate/malate dehydrogenase, putative, expressed | GO:0006108 | malate metabolic process | biological_process | IEA |
| LOC_Os05g49880 | lactate/malate dehydrogenase, putative, expressed | GO:0006099 | tricarboxylic acid cycle | biological_process | IEA |
| LOC_Os05g49880 | lactate/malate dehydrogenase, putative, expressed | GO:0006096 | glycolysis | biological_process | IEA |
| LOC_Os05g49880 | lactate/malate dehydrogenase, putative, expressed | GO:0005975 | carbohydrate metabolic process | biological_process | IEA |
| LOC_Os06g05359 | NBS-LRR disease resistance protein, putative, expressed | GO:0006915 | apoptosis | biological_process | IEA |
| LOC_Os06g05359 | NBS-LRR disease resistance protein, putative, expressed | GO:0006952 | defense response | biological_process | IEA |
| LOC_Os06g29180 | erythronate-4-phosphate dehydrogenase domain containing protein, expressed | GO:0008152 | metabolic process | biological_process | IEA |
| LOC_Os06g29180 | erythronate-4-phosphate dehydrogenase domain containing protein, expressed | GO:0055114 | oxidation reduction | biological_process | IEA |
| LOC_Os06g45510 | thioredoxin, putative, expressed | GO:0045454 | cell redox homeostasis | biological_process | IEA |
| LOC_Os09g36300 | OsLonP4 - Putative Lon protease homologue, expressed | GO:0006508 | proteolysis | biological_process | IEA |
| LOC_Os09g36300 | OsLonP4 - Putative Lon protease homologue, expressed | GO:0006952 | defense response | biological_process | IEA |
| LOC_Os10g33800 | lactate/malate dehydrogenase, putative, expressed | GO:0055114 | oxidation reduction | biological_process | IEA |
| LOC_Os10g33800 | lactate/malate dehydrogenase, putative, expressed | GO:0044262 | cellular carbohydrate metabolic process | biological_process | IEA |
| LOC_Os10g33800 | lactate/malate dehydrogenase, putative, expressed | GO:0008152 | metabolic process | biological_process | IEA |
| LOC_Os10g33800 | lactate/malate dehydrogenase, putative, expressed | GO:0006108 | malate metabolic process | biological_process | IEA |
| LOC_Os10g33800 | lactate/malate dehydrogenase, putative, expressed | GO:0006099 | tricarboxylic acid cycle | biological_process | IEA |
| LOC_Os10g33800 | lactate/malate dehydrogenase, putative, expressed | GO:0006096 | glycolysis | biological_process | IEA |
| LOC_Os10g33800 | lactate/malate dehydrogenase, putative, expressed | GO:0005975 | carbohydrate metabolic process | biological_process | IEA |
| LOC_Os11g07020 | fructose-bisphospate aldolase isozyme, putative, expressed | GO:0006096 | glycolysis | biological_process | IEA |
| LOC_Os11g07020 | fructose-bisphospate aldolase isozyme, putative, expressed | GO:0008152 | metabolic process | biological_process | IEA |
| LOC_Os12g10580 | ribulose bisphosphate carboxylase large chain precursor, putative | GO:0006412 | translation | biological_process | IEA |
| LOC_Os12g10580 | ribulose bisphosphate carboxylase large chain precursor, putative | GO:0015977 | carbon fixation | biological_process | IEA |
| LOC_Os12g27930 | expressed protein | GO:0006486 | protein amino acid glycosylation | biological_process | IEA |
| LOC_Os12g42860 | 2-aminoethanethiol dioxygenase, putative, expressed | GO:0055114 | oxidation reduction | biological_process | IEA |

| **c) Genes unmapped by GO annotation.** | |
| --- | --- |
| **Locus ID** | **RGAP Ver 6 Annotation** |
| LOC_Os04g52920 | remorin family protein, putative, expressed |
| LOC_Os11g39500 | hypothetical protein |
| LOC_Os01g46340 | chloroplast unusual positioning protein, putative, expressed |
| LOC_Os03g32290 | hypothetical protein |
| LOC_Os09g06160 | retrotransposon protein, putative, Ty3-gypsy subclass |
| LOC_Os08g40630 | mTERF domain containing protein, expressed |
| LOC_Os09g31000 | EF hand family protein, expressed |
| LOC_Os05g02530 | glutathione S-transferase, N-terminal domain containing protein, expressed |
| LOC_Os08g18880 | WD domain, G-beta repeat domain containing protein, expressed |
| LOC_Os04g57010 | zinc finger C-x8-C-x5-C-x3-H type family protein, expressed |
| LOC_Os01g11000 | transposon protein, putative, unclassified |

**Supplementary Table S3: Gene loci (38) that were used for GO enrichment analysis (Left Column** )**, and Proteins of which locus IDs were not retrieved with riceDB (Right column)**

| Sr No | **Genes (38) loci used for enrichment analysis with rice array database (**[**http://www.ricearray.org/analysis/go_enrichment.php**](http://www.ricearray.org/analysis/go_enrichment.php)**), for the proteins, of which corresponding Locus IDs were retrieved** | **Proteins, of which corresponding Locus IDs were not retrieved from** [**http://ricedb.plantenergy.uwa.edu.au/**](http://ricedb.plantenergy.uwa.edu.au/) **and therefore not used for GO enrichment** |
| --- | --- | --- |
|  |  |  |
| 1 | LOC_Os12g10580 | A3C0Z2 |
| 2 | LOC_Os11g07020 | B8BFR6 |
| 3 | LOC_Os05g41640 | Q6L4K2 |
| 4 | LOC_Os04g52920 | Q5ZE18 |
| 5 | LOC_Os11g39500 | Q5JKU7 |
| 6 | LOC_Os01g46340 | Q9FW56 |
| 7 | LOC_Os12g42860 | Q67UQ5 |
| 8 | LOC_Os02g33220 | B8B691 |
| 9 | LOC_Os06g45510 | A2WQA2 |
| 10 | LOC_Os01g74650 | B9EUG1 |
| 11 | LOC_Os03g32290 | Q01KS5 |
| 12 | LOC_Os09g06160 | Q5Z6Q9 |
| 13 | LOC_Os04g09430 | B8AMQ0 |
| 14 | LOC_Os03g58270 | Q69YD6 |
| 15 | LOC_Os06g05359 | Q6EQ30 |
| 16 | LOC_Os08g40630 | A2WPN7 |
| 17 | LOC_Os02g57550 | T02B73 |
| 18 | LOC_Os09g31000 | B8B691 |
| 19 | LOC_Os03g24160 |  |
| 20 | LOC_Os02g51470 |  |
| 21 | LOC_Os02g52940 |  |
| 22 | LOC_Os01g31690 |  |
| 23 | LOC_Os09g36300 |  |
| 24 | LOC_Os03g17690 |  |
| 25 | LOC_Os05g02530 |  |
| 26 | LOC_Os04g45580 |  |
| 27 | LOC_Os08g18880 |  |
| 28 | LOC_Os05g49880 |  |
| 29 | LOC_Os02g53270 |  |
| 30 | LOC_Os05g01270 |  |
| 31 | LOC_Os01g46070 |  |
| 32 | LOC_Os02g02870 |  |
| 33 | LOC_Os06g29180 |  |
| 34 | LOC_Os04g57010 |  |
| 35 | LOC_Os03g62060 |  |
| 36 | LOC_Os10g33800 |  |
| 37 | LOC_Os12g27930 |  |
| 38 | LOC_Os01g11000 |  |

**Supplementary Table S4: Antibodies used in this study**

| Antibody | **Abbreviation** | Dilution |
| --- | --- | --- |
| Cyt b559 (9 kDa) | Cyt b559 | 1:1000 |
| Oxygen Evolving Complex (33 kDa) | OEC33 | 1:3000 |
| Oxygen Evolving Complex (23 kDa) | OEC23 | 1:2000 |
| Oxygen Evolving Complex (16 kDa) | OEC16 | 1:4000 |
| Light Harvesting Chlorophyll Protein b1 (25 kDa) | Lhcb1 | 1:500 |
| Light Harvesting Chlorophyll Protein b2 (25 kDa) | Lhcb2 | 1:1000 |
| Cyt f (34 kDa) | Cytf | 1:500 |
| Cyt b6/f Subunit IV (17 kDa) | Subunit IV | 1:500 |
| PSI Subunit III (16 kDa) | PS I F | 1:3000 |
| PSI Subunit IV (11 kDa) | PsaE | 1:4000 |
| PSI Subunit V (17 kDa) | PS I L | 1:3000 |
| PSI Subunit VI (11 kDa) | Psa H | 1:3000 |
| Light Harvesting Chlorophyll Protein a1 (26 kDa) | Lhca1 | 1:4000 |
| Light Harvesting Chlorophyll Protein a4 (16 kDa) | Lhca4 | 1:500 |
